# Small Interfering RNA Carriers for Oncotherapy: A Preclinical Overview

**DOI:** 10.3390/pharmaceutics17111408

**Published:** 2025-10-30

**Authors:** Liliana Aranda-Lara, Alondra Escudero-Castellanos, Maydelid Trujillo-Nolasco, Enrique Morales-Avila, Blanca Ocampo-García, Rigoberto Oros-Pantoja, Virginia Sánchez-Monroy, Keila Isaac-Olivé

**Affiliations:** 1 Laboratorio de Investigación en Teranóstica, Facultad de Medicina, Universidad Autónoma del Estado de México, Toluca 50180, Mexico; larandal@uaemex.mx (L.A.-L.); ealondrac@gmail.com (A.E.-C.); rorosp@uaemex.mx (R.O.-P.); 2 Laboratorio de Toxicología y Farmacia, Facultad de Química, Universidad Autónoma del Estado de México, Toluca 50120, Mexico; emoralesav@uaemex.mx; 3 Departamento de Materiales Radiactivos, Instituto Nacional de Investigaciones Nucleares, Ocoyoacac 52750, Mexico; blanca.ocampo@inin.gob.mx; 4 Escuela Superior de Medicina, Instituto Politécnico Nacional, Salvador Díaz Mirón esq. Plan de San Luis S/N, Miguel Hidalgo, Casco de Santo Tomás, Ciudad de México 11340, Mexico; vsanchezm@ipn.mx

**Keywords:** cancer gene therapy, siRNA transporter systems, cell signaling targets, siRNA therapy

## Abstract

**Introduction**: Gene therapy using siRNA is a current area of research in oncology. Although siRNA formulations have not yet been approved for cancer therapy, numerous studies have demonstrated their therapeutic potential for tumor remission. **Objective**: To provide an overview of the formulations designed and developed to date based on synthetic siRNA for systemic administration to silence cancer genes. **Methodology**: A thorough search was conducted using the keywords “siRNA”, “therapy”, and “cancer”, with further classification of the resulting works into the various topics addressed in this review. **Results**: This review encompasses a wide range of aspects, from the design of siRNA using bioinformatics tools to the primary cellular signals and mechanisms targeted for inhibition in cancer therapy. It describes the primary chemical modifications made to siRNA chains to enhance stability, improve bioavailability, and ensure their binding to nanocarrier systems. siRNA formulations ranging from simple conjugates with biomolecules and small molecules to organic, inorganic, and hybrid nanoparticles, which are examined focusing on their advantages and disadvantages. The significance of nanosystems in dual therapy, including siRNA, for developing personalized treatments that achieve better outcomes is emphasized. **Conclusions**: Personalized cancer therapy appears to be the preferred approach for oncological treatments. To progress, strategies need to be tailored to the patient’s genetic profile. siRNA therapies provide a flexible platform for targeting and inhibiting critical oncogenes, enhancing the prospects of genomics-guided, patient-specific therapies.

## 1. Introduction

Small interfering RNAs (siRNAs) are double-stranded (sense and antisense) non-coding RNA molecules composed of 19–21 nucleotides with two overhanging nucleotides at the 3′ end of the antisense strand. They are naturally generated from a long RNA. They inhibit (silence) the expression of target genes by cleaving complementary messenger RNA (mRNA) into two halves. They are found in eukaryotic cells but can also be obtained artificially. In principle, any gene can be silenced by a siRNA with a complementary sequence, making siRNAs specific and potent drugs for the treatment of diseases. siRNAs also contribute to transcriptional gene silencing by promoting long-lasting epigenetic changes that are faithfully inherited during cell division [1,2,3,4,5]. It has been suggested that two siRNAs, one targeting the promoter and the other targeting exon 1 of the transcript, can strongly repress the target gene. Weinberg and Morris mention examples of mammalian genes transcriptionally regulated by siRNA (e.g., eukaryotic translation elongation factor 1α, nitric oxide synthase, E-cadherin, among others) [6]. 

Treatment with siRNA to silence genes that contribute to tumor growth, survival, metastasis, and resistance to other therapies is very promising. The latter is demonstrated in various clinical trials currently underway [7,8]. Despite these studies, the clinical use of siRNA remains limited. So far, of the six drugs approved for commercial use, five have subcutaneous administration [8]. In oncology, however, systemic administration is more convenient as it is more effective and allows for the treatment of several types of cancer with a single product. As naked siRNA presents difficulties for systemic administration, strategies are continually being developed to protect and transport it to target cells, ensuring cell uptake and intracellular release. This review provides an overview of the general steps involved in the development of siRNA formulations, with a focus on those specifically designed for oncology therapy. It covers the design of siRNA molecules using computer tools, the main chemical modifications to the strands, the production of conjugates and transporter nanosystems, and some of the siRNA target genes currently in clinical research.

Although several recent reviews have been published on the use of siRNA as a therapeutic agent, these primarily describe formulations that have reached the clinical trial phase or are already approved for clinical use [9,10,11]. This work takes another approach. It is an overview that compiles and groups the different non-viral delivery systems that have been designed, prepared, and evaluated regardless of whether they have reached clinical evaluation or not, for cancer therapy. This compilation enables the visualization of the variety of systems and nanosystems that have been explored, as well as the extensive research behind a therapeutic strategy that has reached the clinical evaluation phase.

The keywords “cancer”, “therapy”, and “siRNA” were used to perform the search. The results were then grouped by the different siRNA pathways to reach cancer cells. This review contains a general description of siRNA biological mechanism, the bioinformatics tools for designing synthetic siRNAs, the chemical modifications to siRNA strands, siRNA-biomolecules, siRNA conjugate to small molecules, siRNA nanocarrier systems, multimodal nanocarrier systems for oncology therapy, and examples of key siRNA targets in tumor-promoting pathways and their roles in mono- and combination therapies. A notable feature of this work is that it highlights the primary studies in which siRNA has been investigated as part of multimodal cancer therapies, an emerging area of research. 

## 2. Biological Mechanism of siRNA

siRNA degrades the target mRNA without the need to integrate into the genome or enter the nucleus; it only needs to reach the cytosol to associate with the multi-protein RNA-induced silencing complex (RISC) [11,12]. This complex is a ribonucleoprotein family whose ATP-dependent helicase activity unwinds the siRNA double helix, separating it into its two strands. The core protein of the RISC, Argonaute-2 (Ago2), thermodynamically selects the guide or antisense strand and cleaves the passenger strand into fragments. TRBP and PACT, among other proteins present in the RISC, have been shown to influence guide strand selection [11,13]. Studies suggest that endonucleases such as C3PO can degrade the passenger or sense strand.

C3PO is a Mg^2+^-dependent endonuclease that consists of six Translin/TB-RBP subunits, which confer siRNA-binding specificity, and two TRAX subunits harboring nuclease activity. Evidence indicates that hC3PO functions at the RISC assembly stage rather than in RISC-mediated mRNA cleavage. During this process, the pre-RISC (Ago2/siRNA duplex) is converted into an active RISC (Ago2/guide strand) following Ago2-mediated cleavage of the passenger strand. While the mechanism underlying dissociation of the passenger strand remains unresolved, studies suggest that the intrinsic RNase activity of hC3PO accelerates RISC activation by degrading the Ago2-cleaved passenger strand, liberating the guide strand for specific mRNA recognition and subsequent degradation [14,15].

**Figure 1:**
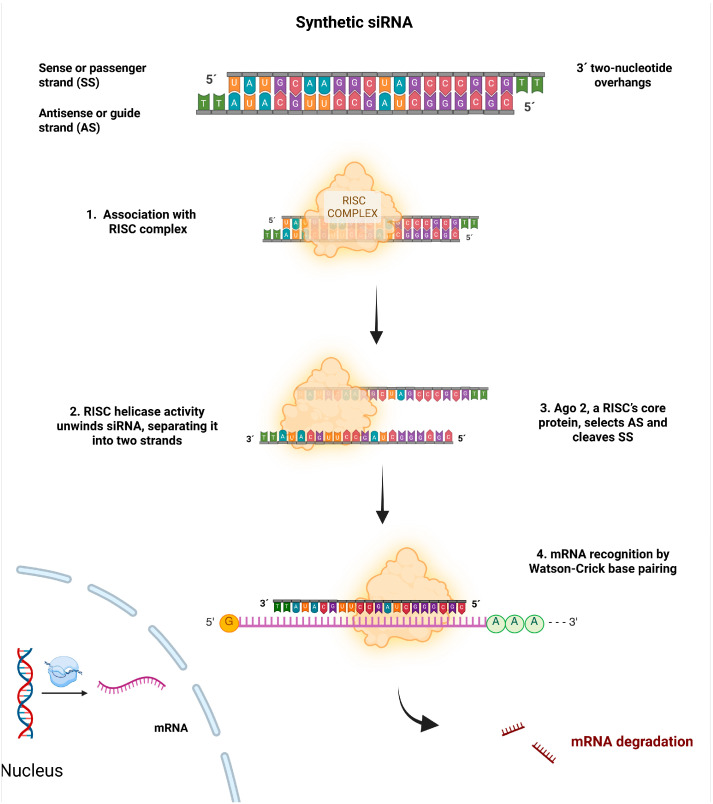
Biological mechanism of siRNA action. The siRNA associates with the RISC, and Ago-2 degrades the sense strand (SS). The remaining antisense strand (AS) serves as a guide to recognize the target mRNA after Argo-2 degrades it. This decreases or suppresses gene expression.

RISC binds to the guide strand at the 5′-phosphate end, recognizes the complementary sequence of the target mRNA by Watson–Crick base pairing. This pairing, which occurs at the position opposite nucleotide 10 from the 5′-end of the guide strand, leads to the cleavage or recruitment of proteins that mediate repression and/or translational mRNA destabilization, via Ago proteins [12,16]. In this way, gene expression is inhibited. A schematic of the process is shown in Figure 1.

## 3. Fundamental Principles for siRNA Design

Gene silencing therapy utilizes rationally designed synthetic siRNAs to degrade complementary messenger RNA (mRNA) selectively. The canonical structure of siRNA consists of a duplex of approximately 19 nucleotides, with two unpaired nucleotides at the 3′ end of each strand, forming two 3′ overhangs [17,18]. Effective siRNA design requires precise optimization of target selection, duplex length, nucleotide composition, sequence specificity, duplex asymmetry, mRNA accessibility, and delivery modality [17,19,20].

The first step in siRNA design is to identify the target gene and select regions within the mRNA that are unique and specific [19,20]. The antisense strand of the siRNA is designed to complement a functional site in the target mRNA. Proper sequence design is crucial for achieving specific gene silencing while minimizing off-target effects. Regarding siRNA length, variations can lead to different outcomes. For example, duplexes longer than 30 nucleotides activate the interferon (IFN) pathway via PKR, causing nonspecific mRNA degradation and apoptosis, which can induce off-target effects [21], whereas duplexes shorter than 15 nucleotides fail to engage the RISC efficiently. 

Considering that the RISC enzyme complex uses the thermodynamic properties of the two strands to decide which strand will serve as the functional guide and which will be discarded, strand design is affected by the thermodynamic asymmetry of the 5′ ends, a principle called the “less stable 5′ end rule” [22]. According to this rule, the strand with lower base-pairing stability at its 5′ end is more likely to be incorporated as the guide, while the more stable strand is chosen as the passenger [13,19,22]. For instance, a duplex with an A–U pair (ΔG ≈ −1.8 kcal/mol, less stable) at one 5′ end and a G–C pair (ΔG ≈ −2.7 kcal/mol, more stable) at the other favors the incorporation of the A–U-containing strand as the guide. This example shows how RISC uses thermodynamic differences to enable efficient gene silencing. For guide strand selection, it is also important that the minimum free energy (MFE) at the 5′ end of the strand is higher than that of the opposite strand, and that the ΔMFE of the first three nucleotides of each strand exceeds 1 kcal/mol. These criteria help identify siRNAs with a higher chance of being incorporated into the RISC [22]. 

**Figure 2:**
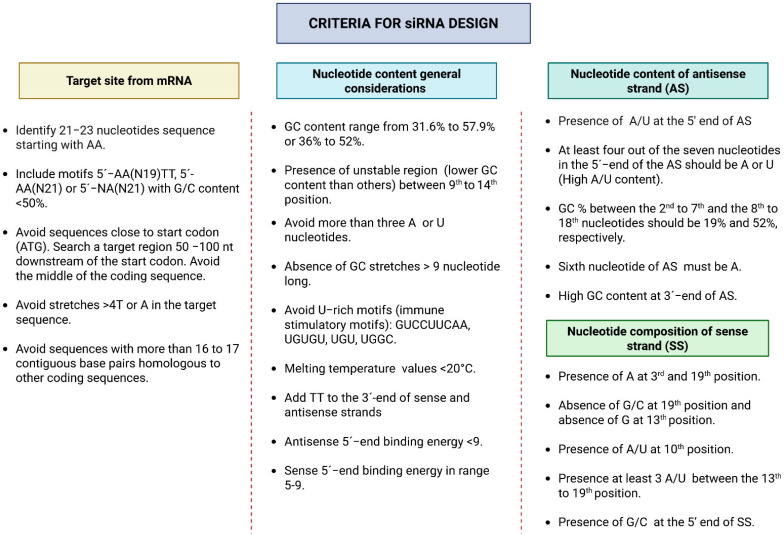
General considerations for functional siRNA design.

The interaction of siRNA with the RISC is heavily influenced by sequence composition and thermodynamic stability. An optimal GC content ranges from approximately 30% to 64%, as too much GC can hinder duplex unwinding, while a region of instability between nucleotides 9 and 14—known as the “energy valley”—helps promote efficient RISC-mediated cleavage [19,23]. Low internal stability at the 5′ end of the antisense strand—fostered by at least four A/U bases within the first seven nucleotides—assists in strand incorporation into RISC. Conversely, high stability at the 3′ end or sequences of G/C bases increase the likelihood of secondary structure formation [19]. Figure 2 illustrates general nucleotide preferences for siRNA design. 

Bioinformatics tools are used to predict potential target sites based on sequence complementarity, thermodynamic stability, and the absence of off-target effects, among other factors. Based on these factors, different variants of molecules are chosen, and possible secondary structures are evaluated. The candidates obtained are experimentally validated to confirm their efficacy and specificity [19,20]. Table 1 shows some of the tools used in the design of siRNAs, as well as their binding directions. 

Tools listed in Table 1 serve different functions. For example, BLOCK-iT™ RNAi Designer is an easy-to-use web tool from Thermo-Fisher that creates RNAi sequences (such as siRNA, shRNA, and miRNA) for any organism using cDNA or GenBank accession numbers. The tool is very effective, with the top three to five designed siRNAs generally achieving over 70% mRNA knockdown under ideal transfection conditions. SMEPred, on the other hand, is a machine learning–based web server that estimates the effectiveness of chemically modified siRNAs. It helps advance siRNA therapeutics by modeling up to 30 chemical modifications, considering both nucleotide composition and modification locations—particularly in the antisense region—to improve silencing efficiency, stability, and minimize off-target effects [27].

Different organizations, such as the RNAi Consortium (TRC) and library manufacturers, offer general standards for siRNA design. There are public databases such as siRNAmod [28], which provide collections of sequences and associated experimental data. This database provides chemically modified siRNAs at various positions, along with experimentally validated permutations and combinations. It also incorporates important information on a specific sequence, including the chemical modification, its respective number and position, structure, simplified canonical input system for molecular data (SMILES), efficacy, cell line, experimental methods, references, and more. 

## 4. siRNA Modifications and Delivery

The main goal in siRNA therapy is to ensure that siRNA reaches the cytosol of the target cell. In vivo siRNA delivery can be administered locally, systemically, or by a combination of both. Local administration (intranasal, intraocular, intrathecal, intratumoral) provides direct access to the target tissue; however, in oncology, the systemic route, particularly intravenous injection, is more convenient as it allows for reaching the target tissue more efficiently. However, naked siRNA is chemically unstable, rapidly cleared by the mononuclear phagocytic system (MPS) and nucleases, exhibits poor cell uptake and endosomal escape, and induces an immune response [12,29]. These drawbacks have led to the development of different delivery strategies, some more clinically viable than others, depending on the tissues to be targeted. The delivery strategies most used are: (i) cell membrane permeabilization by physical methods (beyond the scope of this review), which is little used in the clinic due to its limited effectiveness, although valuable for in vitro studies; (ii) chemical modification of siRNA strands; (iii) developing transport and delivery systems. The latter strategy is the most widely used, mainly with lipidic nanocarriers such as liposomal systems, which are the most prevalent.

### 4.1. Chemical Modifications to siRNA Strands

Early research aimed to improve the specificity, stability in blood, and cell internalization, as well as decrease the toxicity of naked siRNA, focused on chemical modifications to the strands. These modifications depend on the characteristics of each siRNA and the desired target. They are performed on: (i) the rest ribose; (ii) the ribose-phosphate backbone; (iii) the purine and pyrimidine bases. Many of them have been performed to obtain siRNA conjugates with other molecules or to incorporate them into nanoparticles (NPs). The vast majority do not significantly affect the main characteristics of the siRNA [16,30], although some authors have stressed the importance of performing them with great care so as not to compromise the efficacy of silencing and off-target effects [31].

- Modifications to the rest of the ribose. Ribose modifications, particularly at the 2′ position, have been the most widely used to protect siRNA from attack by ribonucleases, improve affinity for the target mRNA, and decrease the immune response. They have the advantage of not altering backbone conformation or gene silencing efficiency. The most common is the substitution of the 2′-OH group with a less nucleophilic group. The most used group is 2′-O-methyl (2′-O-Me), from which derivatives such as 2′-O-methoxyethyl (2′-O-MOE) and 2′-deoxy-2′-fluoro (2′-F) have been developed [32,33,34]. Substitutions can be made at different sites on the double strand or in the central part of the antisense strand [35]. The best results are obtained by alternating 2′-O-Me and 2′-F substitutions [36,37].

Both 2′-O-methyl (2′-O-Me) and 2′-fluoro (2′-F) modifications enhance the chemical and enzymatic stability of siRNA duplexes by stabilizing the 3′-endo conformation of the ribose sugar. This conformation promotes the characteristic A-form helical geometry of native RNA, which is essential for efficient recognition and loading by the RNA-induced silencing complex (RISC). Additionally, the A-form structure provides greater resistance to nucleases, particularly RNases, which typically target the reactive 2′-OH group of unmodified ribose. Of these modifications, 2′-F most closely resembles the electronic and steric properties of the native 2′-OH, ensuring high compatibility with RISC. Strategically placing 2′-O-Me modifications on the passenger strand promotes efficient RISC loading and can further improve strand selection by enhancing thermodynamic asymmetry and duplex stability [38,39,40]. These modifications also mitigate the innate immune response by preventing recognition by toll-like receptors, thereby reducing immune-stimulant effects, such as TNFα production [41,42,43,44]. 

Modifications have also been made to 2′-C and 4′-C, as well as to the complete ribose ring, resulting in siRNAs with various physicochemical and biological characteristics [32,33,34,45]. Most of these modified siRNA molecules start by: (i) connecting the 2′-C and 4′-C positions via a methyl bridge to block the sugar residue at the 3′-C end and form so-called locked nucleic acids (LNAs) whose rigid conformation substantially improves the stability of the siRNA [45,46]; (ii) disconnect the 2′-C and 3′-C positions to form unblocked nucleic acids (UNA) to obtain a wide range of derivatives [45,47,48].

- Modifications to the ribose-phosphate backbone. These are designed to minimize off-target effects and immune responses, enhance cell uptake, and increase the bioavailability of siRNA. The most used is the replacement of a non-bridging oxygen in the phosphodiester group linking two consecutive riboses with a sulfur to form a phosphorothioate (PS) [36,49,50,51], a boron to form a borane-phosphate (PB) [52,53], or an acetate to form a phosphonoacetate [54]. These modifications are used to link the siRNA to different molecules [55]. The most prevalent is the formation of PS, where the sulfur atom preserves the negative charge of the siRNA. The principal benefits of this modification are the improvement of siRNA resistance to nucleases, hydrophobicity, stability, and affinity to plasma proteins, resulting in a longer circulation time [56,57]. However, the number of PS should be limited. This can reduce the silencing effect and induce cytotoxicity [49,58,59]. The best PS substitution variant is at the end of the strands. Some authors have synthesized siRNAs with more than one simultaneous modification, with attractive properties for in vivo use [60]. Alternatives with different phosphate derivatives include phosphorodithioate, PS2 [50,61,62], methylphosphonate [58], 5′-(E)-vinyl-phosphonate [63,64,65]. The phosphotriester groups have also been modified [66].
- Modifications to nucleobases. To a lesser extent than ribose and ribose-phosphate backbone modifications, modifications to uridine, cytidine, and adenosine are made to improve thermal stability, nuclease resistance, cell uptake, and reducing immune response [67,68,69,70]. However, concerns exist about the safety of metabolizing modified siRNAs via this route. The modifications described are used to obtain siRNAs with different structures, conjugates, and nanosystems for transport and delivery. There are numerous recent publications that summarize these modifications and their impact on siRNA properties [9,11,39,71]. Table 2 summarizes the advantages and disadvantages of the modifications described, as well as their applications. A common disadvantage is that, depending on the site and extent of the modification, the physicochemical and biological properties of the siRNA may deteriorate.

Simultaneous modifications to siRNA and its carrier can optimize the nanocarrier system. Such modifications can be achieved through a carefully balanced approach that enhances stability, delivery efficiency, and safety without compromising efficacy. siRNA chemical modifications such as 2′-O-Me, 2′-F, phosphorothioate (PS) linkages, and locked nucleic acids (LNAs) can improve siRNA stability, reduce immune stimulation, and enhance specificity without compromising RNA interference activity. For example, the combination of 2′-O-Me and PS at the termini increases nuclease resistance while maintaining silencing activity [11,72]. Optimization of siRNA-carriers should be engineered to protect siRNA from degradation, facilitate cellular uptake, and promote endosomal escape while minimizing toxicity.

In both cases, siRNA and carrier modifications need to be iteratively screened to monitor safety (e.g., immunogenicity, toxicity) and efficacy (target gene silencing, biodistribution). Preclinical tests should also be conducted to evaluate whether modifications to siRNA or the carrier affect siRNA loading or promote off-target effects. Therefore, experimental designs should be followed, including redesign and continuous evaluation of the effectiveness of the forms. This balanced approach enables the development of siRNA therapeutics with optimized stability, delivery, and biocompatibility, which are essential for clinical success in oncology [73].

### 4.2. siRNA Transporters and Delivery Nanosystems

The most used route for in vivo administration of siRNA is through nanosystems for transport and delivery. These systems are necessary to overcome the primary obstacles associated with the in vivo administration of naked siRNA, namely its high instability and poor pharmacokinetic properties. The phosphodiester bond in siRNA is vulnerable to RNases and phosphatases, which rapidly degrade it and reduce the accumulation of intact siRNA in the desired tissue [11]. These systems can be categorized into two broad groups: viral and non-viral. Viral systems are modified viruses that transport and deliver siRNA into cells. There are several types: (i) adenoviruses; (ii) adeno-associated adenoviruses; (iii) lentiviruses and retroviruses [74,75]. All have high transfection efficiency but have drawbacks such as: (i) limited loading capacity; (ii) off-target effects; (iii) immunogenicity and toxicity. Adeno-associated adenoviruses and lentiviruses infect both dividing and non-dividing cells and are therefore very useful in cancer therapy. Lentiviruses and retroviruses can induce mutagenesis, disrupt essential genes, and activate oncogenes [75]. 

Non-viral systems are more suitable. They have advantages and disadvantages that need to be evaluated in relation to specific therapy. They can be divided into two main groups: (i) siRNA conjugates with biomolecules or small molecules and (ii) nanocarrier systems.

### 4.2.1. siRNA Conjugates with Biomolecules and Small Molecules

Although chemical modifications to siRNA strands improve stability, they may negatively affect cellular uptake, intracellular internalization, and interaction with the RNA-induced silencing complex (RISC), ultimately reducing silencing efficiency. Conjugation to stable and biocompatible molecules significantly improves in vivo efficacy. These conjugates have a simple molecular structure and a well-defined ratio. They allow: (i) improved pharmacokinetic profiles; (ii) tissue-specific targeting; and (iii) improved endosomal escape without affecting silencing capacity, among other advantages [76,77,78]. The production process can be regulated in terms of homogeneity and reproducibility. Depending on the type of molecule to be conjugated and the specific characteristics of each siRNA, binding can be done by covalent or non-covalent bonding. Some conjugates spontaneously form nanoparticles (NPs). Most can be incorporated into carrier NPs of different natures by relatively simple processes.

Although siRNA has four terminal phosphate groups available for conjugation, the groups most commonly used for this purpose are the 5′ and 3′ of the sense strand and the 3′of the antisense strand, previously modified (Section 4.1).

(i) siRNA Conjugates with Biomolecules

**Figure 3:**
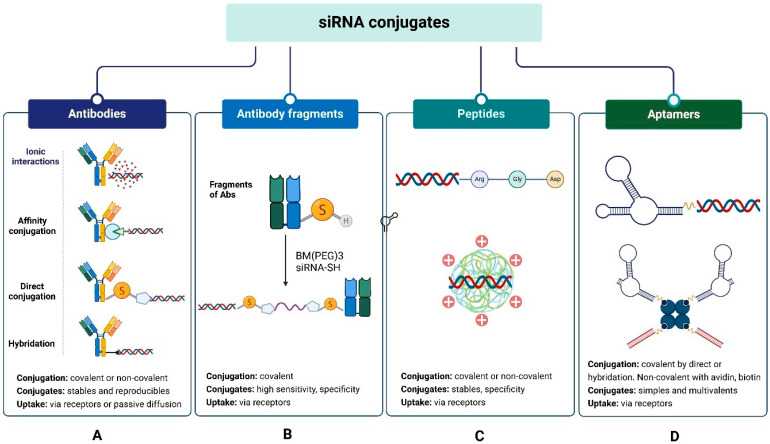
siRNA conjugates with biomolecules: (**A**) antibodies (Ab), (**B**) antibody fragments (Fab), (**C**) peptides, (**D**) aptamers.

This section provides an overview of the main conjugates of siRNA biomolecules. These conjugates have the characteristic that they do not require an additional transport system, as they serve as their own delivery systems. Biomolecule siRNA conjugates consist of the biomolecule, the siRNA, and usually a bifunctional connector for binding. In some cases, spacers are added to improve the stability of the conjugate and prevent the formation of NPs. These siRNA conjugates penetrate the cell through various mechanisms, ranging from passive diffusion to receptor-mediated transport [76]. The most used biomolecules for siRNA conjugation include antibodies (Abs) and their fragments (Fabs), peptides, and aptamers (Figure 3).

- Ab-siRNA and FAb-siRNA conjugates. Abs are ideal carrier systems due to their high affinity and specificity, long half-life in blood, and relatively low immunogenicity. Experience gained with antibody-drug conjugates (ADCs) has enabled the development of antibody-siRNA conjugates (ARCs), which combine the precision of siRNA for target gene silencing with the high sensitivity and specificity of Antibodies for binding to their antigens/receptors on the cell surface. They are an advantageous option for targeting siRNA to antigen-expressing tumor cells because they accumulate and internalize more readily than naked siRNA [76,79,80].

Conjugation of Abs to siRNA occurs by covalent and non-covalent coupling (Figure 3A). Covalent docking is a standard method of linking biomolecules. It can be performed using chemical and biological processes, providing stable and reproducible conjugates; however, it sometimes yields low yields and products with varying Ab:siRNA ratios [80]. Additionally, the upstream modifications required for Ab and siRNA binding are relatively expensive [80,81]. Coupling is performed by two variants: (i) direct conjugation and (ii) conjugation by hybridization of the double strand. Non-covalent coupling is simpler and can be performed in a single step; however, the conjugates are less stable and tend to form heterogeneous aggregates and nanoparticles [82]. It is also carried out by two variants: (i) electrostatic interactions and (ii) affinity bonding. For covalent siRNA-Ab/siRNA-Fab coupling by direct conjugation, different reactions can be used, such as (i) disulfide bridge formation; (ii) thiol-maleimide reaction; (iii) carbodiimide-amine reaction; (iv) amino ring opening and β-lactam; (v) azide-alkyne reaction. The bifunctional connector is usually added to the sense strand of the siRNA, and then the connector-siRNA adduct is linked to the Ab or Fab (Figure 3A,B). The method has the advantage that it allows the use of many standard connectors commonly used to obtain ADCs.

For disulfide bridging, both siRNA and Ab/Fab are modified with thiol groups, and then both molecules react [83]. A variant is to add connectors with thiol groups to Ab/Fab and siRNA and then link the two adducts together [84].

The thiol-maleimide reaction is the most used method for direct siRNA-Ab conjugation [76,85,86]. For this conjugation, the interchain disulfide bonds of Ab (4-linked) are reduced to thiol groups using mild reductants such as dithiothreitol (DTT), 2-mercaptoethanol (2ME), or tris [2-carboxyethyl] phosphine (TCEP). The thiol groups are deprotonated and react, by nucleophilic addition or substitution, with electrophilic connectors without affecting the intrachain disulfide bonds (Figure 3A). The most used connectors are maleimide and its derivatives, including SMCC [45,85], N-succinimidyl 3-(2-pyridyldithio)-propionate (SPDP) [45,85,87], N-succinimidyl S acetylthioacetate (SATA) [85], N-succinimidyl 4-(2′-pyridyldithio) pentanoate (SPP) [87], and N-succinimidyl 3-maleimidopropionate (BMPS) [87]. Peptides have also been used as connectors [88]. The connector first binds to siRNA, and then the siRNA-connector adduct is conjugated to Ab [77,80,86,89]. The main drawback of the reaction is the unspecificity of the binding site. To improve this, cysteine is inserted at specific positions in Ab (via protein engineering). After obtaining the thiol groups from the cysteine, the reaction with the siRNA-connector adduct occurs [82,89]. This variant allows the use of THIOMAB platforms. THIOMABs are Abs with cysteine residues in determined positions. They were developed to obtain ADCs but are also successful for ARCs. When THIOMABs are used, the siRNA is modified to obtain an amine group on the sense strand. This amine group is attached to the amide-thiol linker. Finally, the siRNA-connector adduct reacts with THIOMAB thiols [85]. 

Direct conjugation via the thiol-maleimide reaction can also be performed by first attaching the linker to the Ab and then to the modified siRNA. Under this variant, Ab-siRNA conjugates were produced with cationic gelatin as a linker. Cationic gelatin is obtained by modifying carboxyl groups that bind to lysine residues of Ab through amide bonds. Subsequently, thiol-modified siRNA is conjugated to gelatin [90].

In carbodiimide-amine conjugation, the carboxyl groups either naturally present or chemically introduced into the siRNA are activated with 1-ethyl-3-(3-dimethyl aminopropyl) carbodiimide (EDC). Upon activation, the siRNA forms an intermediate that reacts with primary amine groups on the Ab, typically those on lysine residues, to form a very stable amide bond. The method has the disadvantage that the binding is nonspecific since the linker can also bind to the amine groups of tyrosine and cysteine [89]. Some authors conjugate the EDC to carboxyl groups of Ab and then bind them with siRNA, previously modified with amine groups [80]. The addition of N-hydroxysuccinimide (NHS) enhances the reaction efficiency [91].

Conjugation via the amino ring opening reaction and β-lactam is performed by functionalizing the siRNA with β-lactam. Then, the siRNA-β-lactam adduct reacts with the amino groups of the lysine residues [92]. For direct azide-alkyne conjugation, siRNA is modified with an azide (N3) or an alkyne at the ribose moiety. Ab is conjugated to a linker containing alkyne or azide groups [93,94,95]. The linker can also be conjugated to siRNA [95]. 

Double-stranded hybridization involves covalently conjugating a single-stranded oligonucleotide to the Ab and then adding the complementary strand to form the double-stranded siRNA (Figure 3A) [82]. 

Non-covalent coupling by electrostatic interactions allows for the easy obtaining of Ab-siRNA conjugates with the following advantages: (i) no chemical modification of siRNA is required; (ii) the complex formed is easily internalized and promotes endosomal escape. Cationic peptides that bind siRNA by electrostatic interactions are used, for example, 9-arginines [96] and protamines [97,98,99].

The method consists of first coupling arginine or protamine to the Ab by a covalent bond and subsequently to the siRNA. This spontaneously forms a peptide-siRNA complex (Figure 3A). The positive charges of the 9-residue of arginine and protamine interact with the negative charges (~40) of siRNA phosphates. For covalent binding of siRNA to arginine or protamine, bifunctional linkers such as those mentioned are used (e.g., SMCC) [97,98,99]. DPDPB (1,4-di-(3′-[2′pyridyldithio]-propionamido) butane) is also used [80]. The choice of linker depends on the coupling site. EDC, for example, couples the carboxyl groups of the Ab to the amine groups of 9-arginine, and SPDP couples the thiol groups of the Ab to the amine groups of 9-arginine. If a 9-arginine derivative with a cysteine residue is used, then its thiol groups can be coupled to the thiols of the Ab using DPDPB as the linker [80]. SMCC is used to couple the amino terminus of protamines to the thiol groups of Ab [97,98,99]. There are commercial kits that allow these couplings to be made easily.

Ab-siRNA electrostatic complexes have been used to transfect siRNAs into different cell types. However, the method has two significant drawbacks: (i) depending on the reaction conditions, aggregates and superstructures can form [99]; (ii) since the interactions are reversible, they can dissociate under certain pH and salt concentrations [82].

Non-covalent affinity coupling (Figure 3A) is based on spontaneous affinity reactions between avidin and biotin [100,101]. It has the advantage that it does not require purification. Some authors, however, consider that it is not helpful for therapeutic applications.

Avidin-biotin conjugates can be obtained by (i) modifying the siRNA with a thiol to conjugate it to the previously maleimide-linked avidin. Biotin is also modified and binds to the primary amines of the Ab. The siRNA-avidin adduct is then bound to the biotinylated Ab [100,101]; (ii) linking the Ab to avidin and the siRNA to biotin, using tetraethylene glycol (TEG) as a connector [79,100]. Since avidin has four binding sites for biotin, some authors biotinylate both the siRNA and the Ab and subsequently bind them to avidin [89]. The high affinity between avidin and biotin (K_D_ = 10^−15^ M and t_1/2_ (disoc) = 89 days) [101] enables the formation of conjugates with high stability [80,101]. Similar methods obtain Fab/siRNA conjugates.

Ab-siRNA/Fab-siRNA conjugates present several limitations: (i) in some cases, multiple tissues express the same antigens and/or cell receptors, leading to potential nonspecific targeting; (ii) some conjugates exhibit low levels of internalization, particularly in solid tumors; (iii) others may interfere with the immune system or induce toxicity [78,80,82,89].

- Peptide-siRNA conjugates. The small size of peptides, their relatively low molecular weight, ease of synthesis, and low cost of production have facilitated the development of peptide-siRNA conjugates (Figure 3C) with lower immunogenicity and toxicity, better pharmacokinetic properties, high cellular uptake, and more efficient endocytosis than Ab-siRNA conjugates [102,103]. Amino acids (AA) with acidic, hydrophilic, hydrophobic, or aromatic residues can be combined to generate several peptides with siRNA delivery potential, called cell-penetrating peptides (CPP) or membrane transduction peptides (MTP). These peptides are generally composed of <30 AA, often including lysine, histidine, and arginine. They readily cross the anionic surface of cell membranes and reach intracellular compartments without interacting with receptors or altering their functions [102,104,105]. They can be chemically modified to enhance endosomal escape and decrease the influence of endocytic proteases [105,106]. They have been used to internalize, through passive diffusion or endocytosis, various types of macromolecules, including siRNA. Some are internalized by both mechanisms, depending on factors such as the AA sequence and its structure, the CPP/siRNA concentration ratio, cell line and others [102,107,108].

The first CPPs used in siRNA delivery were natural peptides such as (i) the gene transcription transactivator (TAT, 49–57), with high arginine content, derived from HIV-1 TAT protein and (ii) penetratin, peptide derived from the third helix of the *Antennapedia homeodomain* [109,110,111]. The key functional component of these peptides is the guanidine group of arginine, which is capable of forming hydrogen bonds with various elements of the cell membrane [104,112]. Some chimeric and synthetic peptides can interact with cell membrane phosphates, carboxylates, and phosphonates [104]. Among the most used CPPs is a chimeric peptide of 27 AA in length that contains 12 functional AA from the amino terminus of galanin and mastoparan at the carboxyl terminus, connected through a lysine [108,113], as well as low molecular weight protamine derivatives (LMWP) [107,114]. Currently, many CPPs with different AA sequences are available, which differ in length, charge, hydrophobicity, structure, and flexibility. These peptides can penetrate a wide variety of cells, with low toxicity and high transfection efficiency.

Some CPPs also enhance endosomal escape and increase the accumulation in the cytosol of the peptides captured by endocytosis and remain trapped in endosomes for a long time. They are referred to as endosome-disrupting peptides [104,110,115]. These peptides are obtained by introducing histidine residues into the CPPs, which destabilize the endosomal membrane due to their sensitivity to pH changes [116,117]. They are especially lethal to drug-resistant cells, making their application in combination therapies beneficial [115]. However, they require careful evaluation of the balance between endosomolytic activity and toxicity.

Other CPPs are modified to specifically target tumor cell-associated receptors (integrin, somatostatin, gastrin, neurotensin, folate receptors, and others). They are then called targeted peptides. The delivery of siRNA with targeted peptides enhances cell uptake and internalization [118,119,120]. In some cases, they do not require additional agents for transfection [110]. Among the most studied targeting peptides are the cyclic peptides Arg-Gly-Asp (RGD) [121,122]. The RGD receptor, integrin alpha V/β, is ubiquitously expressed on tumor endothelial cells, making it a very favorable target. Some CPPs combine endosomal escape and uptake via receptors and are therefore referred to as multifunctional CPPs [123].

Like Abs, peptides are coupled to siRNA by covalent and non-covalent bonds. The choice of one or the other method depends on the sequence and modification of the side chains of the AAs that make up the peptide, as well as its structure. Conjugation by covalent bonds produces better-defined conjugates that are very stable in circulation [114,121,124]. It is usually performed by direct conjugation, in a similar manner to that described for direct Ab-siRNA conjugation. The most common method is the thiol-maleimide reaction, which involves the amine of the lysine or cysteine residue in the peptide [114,124,125]. The previously modified peptide is bound to the amide-thiol linker, and subsequently, the peptide-linker adduct is reacted with the siRNA, which is also modified with thiol groups. The conjugates obtained are monomeric [114,124,125] and easily release siRNA into the cytoplasm because the disulfide bridges that are formed are easily reduced in this environment [114,124].

When peptides with a high cationic charge are used, it is recommended to place spacers between the peptide and the connector to prevent the interaction between positive charges and negative charges of siRNA, and to form aggregates [111,124,125]. The addition of spacers has been shown to improve cell internalization compared to systems without spacers. One of the most used spacers is PEG, which also contributes to reducing the immune response [102,114]. 

Another strategy for covalently linking peptides to siRNA is the azide-alkyne reaction. The AA sequences of the peptide are modified to decrease positive charges, a crucial step in preventing aggregation. Then, an azide group is introduced into the terminal chain of the peptide. The siRNA is modified with an alkyl group and finally reacts with the azide-modified peptide. The inertness of the alkyne and azide towards other functional groups, such as amines, carboxylates, thiols, alcohols, and esters, makes the binding very selective and regiospecific. The reaction is also insensitive to oxygen and water. It allows the preparation of conjugates with high stability in serum but requires an additional transfection agent [94,126,127]. The reaction is also used to obtain peptide-siRNA conjugates by hybridization [128]. 

Similar to Ab-siRNA conjugates, covalent peptide-siRNA conjugation requires many steps and a high level of purification to exclude the effects of the free peptide. Furthermore, the formation of covalent bonds between the peptide and siRNA can affect their biological activity [78,108,127]. Some conjugates may remain trapped in the endocytic pathway, while others trigger immune responses.

Obtaining peptide-siRNA conjugates by non-covalent bonds is simpler. Electrostatic interactions primarily drive it. To form these conjugates, low-molecular-weight cationic peptides with a high charge density of lysine or arginine (+7 to +10) are used. Among the most used are octaarginine (R8), nonaarginine (R9), and their derivatives [120,125,129,130]. The drawback of the method is that to obtain an appropriate conjugate that easily penetrates the cell, an excess of peptide is required. This can lead to the formation of unwanted aggregates with complex secondary and tertiary structures [120,125], and increase conjugate toxicity [108,116,131]. Some tend to be localized in the nucleus rather than the cytosol [124].

AA sequence and peptide structure are significant factors in the formation of electrostatic peptide-siRNA conjugates. Some authors consider that the best way to obtain stable electrostatic conjugates is to use amphipathic peptides [116,125,132,133]. The peptide’s cationic part forms an electrostatic complex with siRNA, and the hydrophobic part contributes to structural stability and cell internalization [134]. Amphipathic peptides with high silencing capacity and low toxicity can be obtained by the addition of tryptophan (aromatic) residues at one end and arginine and lysin [132,135]. However, there is evidence that amphipathic peptides tend to produce off-target effects and toxicity [134].

Although siRNA-polypeptide conjugates are typically delivered using carrier systems rather than conjugates, polypeptides are included here due to their structural similarities with peptides. siRNA-polypeptide conjugates face several challenges: (i) they interact with blood proteins and form aggregates that accumulate in the lungs, liver, and spleen [29,136]; (ii) they tend to induce off-target effects [137]; (iii) some exhibit low encapsulation efficiency [138,139,140]; (iv) others fail to promote efficient endosomal escape or cause toxicity [29,140]. To address these issues, various strategies have been developed, such as (i) modifying the surface of the siRNA-polypeptide conjugate by coating with PEG [141,142] and/or conjugating with molecules that target cell receptors [143,144]; (ii) incorporating the conjugate into inorganic or organic nanostructures to create hybrid nanosystems; (iii) adding histidine and arginine residues; histidine allows pH-responsive siRNA release and endosomal escape [145], while arginine enhances membrane penetration via receptor-mediated endocytosis [146]; (iv) improving siRNA release by attaching non-cationic components to the cationic carrier, which triggers charge conversion to reduce binding affinity at the target site, such as the cytosol [147]. One of the most used polypeptides is polylysine (PLL).

PLL-siRNA conjugates employ PLL, a widely used polypeptide for siRNA delivery. They are characterized by biocompatibility and biodegradability; however, they tend to exhibit instability in vivo and may pose toxicity concerns [148,149]. The facile chemical modification of PLL facilitates the creation of conjugates with various spatial configurations [149,150,151], which can be incorporated into alternative nanosystems [139,148]. Conventionally, PLL is modified through the addition of histidine and arginine residues [152].

- Aptamer-siRNA conjugates: Aptamers are single-stranded DNA or RNA molecules known for their high affinity and specificity for cell receptors overexpressed in tumors. Their large-scale production and low cost have driven the development of aptamer-siRNA conjugates (Figure 3D), also known as chimeras, as alternatives to Ab-siRNA and peptide-siRNA conjugates [153,154]. Unlike antibodies and peptides, aptamers maintain their biological activity when linked with siRNA. Since aptamers are smaller (6–30 kDa) compared to antibodies (150 kDa), their conjugates with siRNA are more easily internalized, and their endosomal escape is more efficient [155,156,157].

The broad chemical versatility of aptamers enables the synthesis of multivalent and simple aptamer-siRNA conjugates through both covalent and non-covalent interactions [153,158]. Covalent attachment is accomplished via direct methods or hybridization. In both methods, the aptamer and siRNA strands are modified, and then linked through reactions such as: (i) carbodiimide; (ii) thiol-maleimide; (iii) azide-alkyne, as previously described [158,159,160]. Some researchers also use a technique called oxidative coupling [153]. 

The hybridization process is similar to that used for Abs and CPPs. It begins with in vitro transcription to produce a siRNA strand bound to the aptamer, followed by the addition of the complementary strand [157,161,162]. Another method involves the so-called “universal adhesive bridge” conjugation, where the 3′-end of the aptamer is joined to the 5′-end of the sense strand of the siRNA via a U-U-U connector acting as a bridge. The complementary strand of the siRNA is then added (Figure 3D) [163,164,165].

An advantage of hybridization conjugation is that multivalent siRNA-aptamer conjugates can be obtained. For example, two different siRNAs can be linked with a bivalent aptamer to simultaneously silence two different genes [166,167]. Multimeric conjugates called comb-type conjugates can also be obtained [155,160]. These conjugates are formed by the hybridization of antisense chains of the siRNA with sense chains that are previously bound to the aptamer (Figure 3D).

Aptamer-siRNA conjugates by covalent means are expensive [168], so many authors prefer non-covalent binding by affinity conjugation using the avidin-biotin system [159,165]. The siRNA binds to biotin and the aptamer to avidin (or vice versa). Similarly, two biotinylated siRNAs and two biotinylated aptamers can be used and bound to avidin [169]. To ensure the release of siRNA into the cytosol, a cleavable linker can be added between the siRNA and the biotin. This allows the preparation of multivalent conjugates with a greater internalization capacity compared to the usual conjugates, although silencing does not improve significantly [168,169].

Aptamers are easily degraded in the biological environment. To increase the blood circulation time and silencing efficiency, different strategies are used such as: (i) truncating the aptamer [161,170]; (ii) adding overhanging nucleotides to the antisense strand of the siRNA [161,171]; (iii) add PEG to the sense strand of siRNA [161,170]; (iv) mixing (i)–(ii) [169]; (v) link the aptamer-siRNA conjugate to nanocarrier systems of different nature. The latter is one of the most widely used strategies [155,156,158,166,172].

- Toxins-siRNA conjugates. Toxins are poisonous substances produced by living organisms such as plants, animals, and microorganisms. The AB-type toxin has a domain that can be engineered to reduce (attenuate) toxicity, a translocation domain that enables endosomal escape, and a receptor domain that induces endocytosis mediated by cell receptors [173]. Diphtheria and anthrax are examples of AB-type toxins, which have been used to create conjugates for delivering siRNA in vitro to cancer cells [173,174]. siRNA conjugates with attenuated AB-toxins become a promising delivery system because they address some of the challenges of siRNA delivery, especially endosomal escape, and improve upon some disadvantages of polycationic delivery systems. Although these engineered modified toxins have been used for protein delivery [175], few studies have reported on siRNA delivery. This field is likely to grow in the future as methods for modifying toxins and conjugating siRNA continue to advance.

(ii) siRNA Conjugates with Small Molecules

The binding of siRNA to small molecules has led to the formation of conjugates with low toxicity and immunogenicity, which can easily penetrate the tumor microenvironment [39,55]. Among these molecules, the most used in oncology therapy are folic acid (FA), cholesterol, fatty acids, and calcium complexes. These molecules can be conjugated directly to the siRNA or used in a packaged delivery vehicle. Anisamides and α-tocopherol have been used to a lesser extent. N-acetyl galactosamine (GalNAc) siRNA conjugates, which are widely used in clinical practice, are only mentioned here as they are not used in oncology. These conjugates with small molecules employ different targeting mechanisms, such as receptor recognition or enhancing cell internalization through hydrophobicity.

Conjugating siRNA to small molecules has been achieved by direct covalent binding. Copper-catalyzed azide-alkyne cycloaddition (CuAAC) is one of the most widely used routes [176,177]. The reaction is based on modifying the sense strand of the siRNA with one or more alkyne groups that are introduced either at the ends or in central positions. The small molecule is modified with the azide and attached to a linker. Depending on the molecule to be linked, hydrophobic or hydrophilic azides are used. Finally, the parts are reacted to form the conjugate. The reaction can be carried out either in the liquid phase or on solid supports, allowing the molecule to be conjugated to the siRNA with simple purification. This reaction is widely used to obtain RNA conjugates with folic acid (FA), cholesterol, long-chain fatty acids, oligoamines, and carbohydrates [176,177,178]. Some siRNA conjugates with small molecules are listed below.

- FA-siRNA conjugates. FA is a crucial component for cell growth and proliferation, serving as a transporter for several therapeutic agents. It is especially useful in oncology because FA receptors (FR), particularly FRα and FRβ, are overexpressed in many tumor cells, while they are expressed at very low or no levels in normal tissues [179,180]. The uptake of FA via FR is specific and exhibits high affinity (K_D_ 10^−9^ M) [181]. The small size of FA-siRNA conjugates allows them to easily reach solid tumors [179,180]. These conjugates are produced by direct covalent conjugation or by hybridization [177]. However, direct conjugation has limitations. Since folate conjugates bind to FRs through the pteridine residue, conjugation must occur through the glutamate residue, which, having two carboxyl groups, results in mixtures of α and γ isomers [182]. Although folate can be incorporated into the 3′ or 5′ ends of the sense strand of siRNA, many researchers prefer central positions [179]. Common covalent conjugation methods include: (i) linking siRNA to a bifunctional amide thiol linker followed by attachment of folate [181]; (ii) connecting the 5′-end of an oligodeoxynucleotide (ODN) to a folate molecule (ODN-FA), then extending the sense strand of the siRNA to couple it with the ODN-FA adduct [183]; and azide-alkyne reactions with various variants [177,184]. The same reactions are used for conjugation via hybridization [183].

For these covalent FA-siRNA reactions, a wide diversity of linkers/spacers is available. For example, the most used are PEG, NHS, N, N′-dicyclohexylcarbodiimide (DCC), polyethylene imine (PEI), N-propargyl-diethanolamine, and dibenzo cyclooctyl amine (DBCO) [179,181,184].

- Cholesterol-siRNA conjugates were developed to increase hydrophobicity and improve siRNA cell uptake [185,186,187]. They are created through direct covalent bonding or hybridization. The most commonly used reactions are thiol-maleimide and azide-alkyne. For the linkage, the 3′ or 5′ ends of the sense strand are modified, followed by adding cholesterol bound to a linker [188,189,190]. The common reaction methods include: (i) attaching the cholesterol connector to a terminal -OH group of the sense strand of the siRNA [188]; (ii) performing PS modifications at the 3′-end of both strands, adding modified nucleotides (2′-OMe) to the antisense strand, and attaching the cholesterol linker to the sense strand [188,190]; (iii) similar to the previous method but alternating modifications with 2′-OMe and 2′F, then attaching nucleotides to the antisense strand [191]; (iv) truncating one or both strands to produce short asymmetric siRNAs, then adding nucleotides with PS modifications to the antisense strand and attaching the linker-cholesterol adduct to the sense strand [189]. These variants enhance thermodynamic stability, pharmacokinetics, internalization, and silencing efficiency. The linker’s nature and the siRNA binding site significantly influence the biological properties of the conjugates [185,189,192]. The most mentioned connectors include triethylene glycol (TEG) [190,192], 2-amino butyl-1-3-propanediol (C7) [188], trans-4-hydroxyprolinol [78,190], and hexamethylenediamine [189].

siRNA-cholesterol conjugates tend to remain trapped in the cell membrane. To improve internalization, so-called tagged siRNA has been obtained. A tagged siRNA is obtained by chelating the siRNA with six cholesterol conjugates. Each cholesterol conjugate is formed by a chelator, a ligand, and a cholesterol molecule. One of the cholesterol conjugates is captured by the cell membrane, and the remaining five “push” the siRNA inwards. The pushing force disappears when the siRNA is released into the cytosol. Ethidium [193] and zinc (II)-dipicolylamine (Zn/DPA) [194] are used as chelators.

The application of cholesterol-siRNA conjugates is primarily carried out locally (in the skin, eyes, and brain [185,195]. The application in oncological therapy (and by systemic route) is used less since a large percentage (80%) accumulates in the liver [196] and in organs that overexpress LDL/HDL receptors [77,185,189]. Some conjugates can also induce cytotoxicity at high concentrations [196,197]. For these reasons, cholesterol-siRNA conjugates are preferred for incorporation into lipid nanosystems for oncology therapy.

- Fatty acid conjugates with siRNA. siRNA has been conjugated to fatty acids such as docosanoic acid (DCA, C_22_H_44_O_2_), docosahexaenoic acid (DHA, C_22_H_32_O_2_), lithocholic acid (LCA, C24H40O3), palmitic acid (C_16_H_32_O_2_), among others [198,199]. These conjugates are obtained by methods similar to those described for cholesterol-siRNA conjugates. Their physicochemical properties, biodistribution, and silencing capacity depend on the specific characteristics of each fatty acid [198,199,200,201,202]. However, their direct use is also limited. For this reason, it is preferred to associate them with lipid nanosystems.
- Calcium-siRNA complexes. The spontaneous formation of calcium ion with siRNA produces nanocomplexes that are stable, have a uniform size (~100 nm), and carry a negative surface charge (−8 mV). The reversible nature of the electrostatic interactions between Ca^2+^ and siRNA has been effectively used for in vitro siRNA transfection [203]. These complexes serve as the foundation for developing highly efficient siRNA nanocarrier systems [204].
- Anisamide-siRNA conjugates. One and two receptors are polypeptide chains located in the endoplasmic reticulum and as transmembrane proteins in nervous system cells. They have neuromodulatory and ion-channeling functions and bind to a wide range of psychoactive drugs [205]. These receptors are also expressed in several tumor cell types [205] and exhibit high affinity for anisamide (2-(4′-methoxy benzamido) ethyleneamide) and its derivatives [206]. Anisamide conjugates with drugs demonstrate high internalization efficiency via endocytosis mediated by these receptors [207,208], which has driven the development of mono- and multivalent anisamide-siRNA conjugates. Monovalent conjugates are formed by direct covalent bonding between an anisamide phosphoramidate and the 5′ end of the sense strand of the siRNA, which has been previously modified with 2′O-Me. For multivalent conjugation, multifunctional linkers are used to first bind to the siRNA and then to the modified anisamide. These conjugates show a high transfection capacity in prostate cancer cells [206]. However, the use of anisamide-siRNA conjugates remains limited, likely due to concerns about potential side effects. Some have been incorporated into nanocarrier systems.
- N-acetyl galactosamine-siRNA conjugates. These are designed to silence specific mRNAs of liver proteins by binding to asialoglycoprotein receptors ASGP-R [209,210]. These conjugates consist of three molecules of N-acetyl galactosamine (GaINaC) linked via a spacer to the siRNA. By using different spacers and modifications of the siRNA, a wide variety of these conjugates have been developed, and they are incorporated into nanocarrier systems. Some of these formulations have received approval for treating various diseases [8].

Other small molecule siRNA conjugates used in oncology therapy include anandamide-siRNA for immune cell treatment [211] and α-tocopherol-siRNA for liver disorders and pancreatic cancer therapy [212,213].

Although small-molecule siRNA conjugates provide a simple and promising method for targeted delivery, especially to specific tissues like the liver (e.g., GalNAc–siRNA), many of these conjugates show poor pharmacokinetics, which restricts their effectiveness in vivo. These issues include low stability, inadequate delivery to target tissues, and potential off-target effects. To overcome these problems, small-molecule siRNA conjugates are often incorporated into nanocarrier systems. Although more complex, nanocarriers offer improved stability, better biodistribution, and the ability to co-deliver therapeutic agents, making them a more versatile and effective platform for a broader range of therapeutic uses.

### 4.2.2. siRNA Nanocarrier Systems

Although modifications to the strands and conjugations with biomolecules and small molecules help avoid many problems linked to naked siRNA, nanocarrier systems are the best choice for systemic siRNA delivery in cancer therapy. These nanocarriers are tiny structures (NP) that have a therapeutic effect similar to or greater than free drugs, with fewer side effects. 

The essential physicochemical properties to consider in a nanosystem for transporting therapeutic agents (including siRNAs) are size, shape, surface charge, hydrophobicity, and surface modifications [214,215]. Emerging evidence indicates that mechanical properties such as deformability, stiffness, and elasticity are equally important in affecting biological outcomes [216,217]. Sizes larger than 10 nm are preferred because they are not easily eliminated by the kidneys, and sizes less than 200 nm are favored because they can penetrate the tumor neovasculature through the fenestrations of endothelial cells in tumor blood vessels (EPR effect). A size smaller than 200 nm also prevents them from being cleared from the bloodstream (which could lead to accumulation in the liver and spleen and activate the immune system) [218], allowing them to accumulate in the tumor. 

Describing the ideal nanocarrier can be challenging. Usually, their size is around 100 nm, especially for rigid nanoparticles like inorganic ones and some polymeric variants. Soft particles larger than 200 nm, such as lipid nanoparticles, micelles, liposomes, and certain polymers, can be internalized by tumors because they are capable of deforming and flattening. However, these bigger particles are more susceptible to clearance from the bloodstream. Spherical shapes are generally preferred for improved cellular uptake [219], although they tend to activate the immune system [220]. For longer blood circulation, neutral or negatively charged surfaces at physiological pH are ideal, as they minimize nonspecific serum protein interactions and off-target tissue uptake, thereby increasing specificity [214,215]. Nonetheless, membrane electrostatic repulsion makes their internalization into target cells less efficient [220]. Additionally, the nanoparticle pKa affects off-target organ uptake in systemic siRNA therapy, with an optimal pKa around 5.4–6.5 to ensure effective endosomal escape. In acidic endosomes, neutral or anionic nanocarriers become protonated, which disrupts membranes and releases siRNA into the cytoplasm [221].

For siRNA transport, the overall surface charge of the carrier depends on the chemical state of the siRNA. If naked siRNA is transported on the nanoparticle surface, the nanoparticle should have a positive charge. Conversely, if siRNA forms a complex, it can be transported inside or on the surface of a neutral or negatively charged nanoparticle. Nanoparticles with hydrophilic surfaces are preferred to reduce the corona effect and are often coated to improve colloidal stability [214,215]. On the other hand, hydrophobic domains stabilize nanoparticles and facilitate cellular uptake, also decreasing toxicity; however, they may increase the corona effect [16]. The nanoparticle surface should be modified with a molecule that enables specific recognition and targeting to the tumor site.

Generally, the relationship between nanoparticle size and immune response is as follows. Nanoparticles smaller than 500 nm are most effectively recognized and taken up by dendritic cells and macrophages. Specifically, those between 100 and 200 nm are internalized via endocytosis, which triggers CD4 and CD8 responses as well as Th1-type immune responses (cellular-based immunity). These smaller nanoparticles elicit a stronger immune response than larger ones. Particles larger than 500 nm are absorbed through phagocytosis and micropinocytosis by macrophages specialized in taking up bigger particles. They stimulate a humoral adaptive response (antibody-mediated immunity) [222].

All of the above factors must be considered when designing siRNA nanocarrier systems. Although the mentioned characteristics improve tumor biodistribution and accumulation, each must be studied thoroughly for every carrier used. Nanocarrier systems are classified into two main categories: “organic” and “inorganic and hybrid”. The choice between them depends on the specific application. Each type has its advantages and disadvantages that influence silencing effectiveness.

(i) Organic Nanocarriers

**Figure 4:**
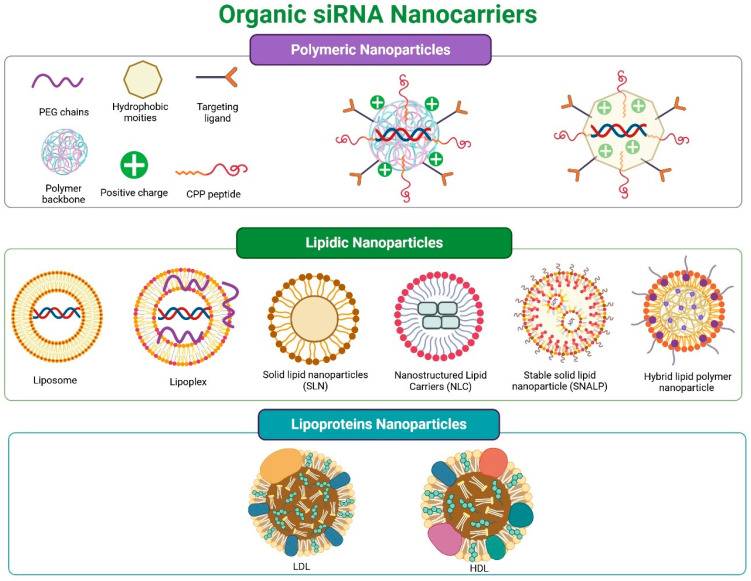
Organic siRNA nanocarriers: (top panel) polymeric; (middle panel) lipidic: lipoplexes, liposomes, solid lipid nanoparticles (SLN), nanostructured lipids (NLC), stabilized nucleic acid lipid particles (SNALP), lipid-polymer hybrid nanoparticles; (bottom panel) lipoproteins: LDL and HDL.

Organic nanocarriers are derived from natural, synthetic, or semi-synthetic sources. To date, the most studied siRNA transporters are polymeric and lipid-based (Figure 4). Cell derivatives have been examined to a lesser extent. Some authors include carbon derivatives in this group, but in this work, they were classified as inorganic nanocarriers.

(a) Polymeric Nanosystems

Polymeric nanosystems carrying siRNA (polymeric NPs) consist of polymers and siRNA, which may be attached to other molecules and include auxiliary components (see Figure 4 (top panel)). These NPs are highly durable, exhibit low polydispersity, and possess high stability. They are easy to customize for various applications and are cost-effective [223,224]. They can be cationic, anionic, or amphiphilic, whether natural or synthetic, but all share qualities of biodegradability, biocompatibility, and low immunogenicity [224,225].

Among the natural polymers most used in siRNA transport, chitosan (CS) stands out [141,226,227]. To a lesser extent, alginates, cellulose, gelatin, atelocollagen, and hyaluronic acid have been used [228,229]. The diversity of synthetic polymers is extensive and complex to cover. Among the most used are polyethylene imine (PEI) and poly(β-amino esters) (PBAE) [136,148,149,230,231]. Polylysine (PLL) can also be considered as a synthetic polymer, but in this work, it was included in the previous subsection “Peptide-siRNA conjugate”, because it is also a polypeptide.

A useful way to classify polymeric nanosystems is based on the morphology of the nanoparticle formed. Polyplexes are created from cationic polymers (e.g., PEI, CS, PBAE). A polyplex is a complex formed through spontaneous electrostatic interactions between the polymer’s positive charge and the negative charge of the siRNA. Nanogels, polymersomes, or polymeric micelles are formed from amphiphilic polymers. A positively charged polymer binds to siRNA, and hydrophobic residues compact this polyplex within the nanosystem. Nanospheres are primarily formed through self-assembly of hydrophobic polymers and copolymers (e.g., PLA, PLGA, and PCL), where hydrophobic segments aggregate to create an inner core, while hydrophilic portions form a stabilizing outer shell in aqueous environments. PLGA (poly-L-lactic-co-glycolic acid) is a specific anionic polymer that is “cationized” by adding functional groups, mainly positively charged amines. All these structures are mostly spherical [223,225,232,233], which carry siRNA in a condensed or complexed form [137,138,139,223,234].

The physicochemical and biological characteristics of polymeric carriers are influenced by factors such as molecular weight, charge density, and the spatial configuration of the polymer (whether linear or branched). Additional determinants include the size of the siRNA, ionic strength of the preparation medium, concentrations of both polymer and siRNA, the ratio between the amines of the polymer and the phosphates of the siRNA (N/P ratio), order of reagent addition, among others [16,29,223,225,232]. Multiple techniques are employed for synthesis, including emulsification, nanoprecipitation, ionic gelation, and the use of supercritical fluids [139,232]. Depending on the chosen preparation method, siRNA can be encapsulated within the core, entrapped within the polymeric matrix, covalently conjugated to the polymer, or adsorbed onto the surface. This allows for the utilization of both naked and conjugated siRNA [139,232] 

Cationic polymers are the most commonly used in polymeric nanoparticles for siRNA delivery [29,143,223,235,236]. These polymers can be surface-modified, being linked to peptides, receptor ligands, polysaccharides, exosomes, or cancer cell membranes. Such modifications aim to enhance siRNA delivery to cancer cells. A recent review covers these modifications [237]. In this work, only the most representative polyplexes—PEI-siRNA, CS-siRNA, and PBAE-siRNA—are described below.

- PEI-siRNA polyplexes. PEI is a widely used cationic polymer which forms polyplexes with siRNA because of its high cation density and ability to buffer protons across a broad pH range [238,239]. Its structure includes repetitive ethylenimine groups, which confer extensive buffering capacity within the pH range of the endosomal/lysosomal pathway [240,241]. Chemically, it is highly versatile, easily functionalized and branched [136,240,242]. The transfection efficiency of PEI-siRNA polyplexes is high because, under acidic conditions, their amines facilitate endosomal escape, helping siRNA reach the cytoplasm [136,241]. Branched PEI generally outperforms linear PEI in transfection efficiency [241,243,244]. Additionally, some PEIs can form covalent complexes with siRNA [241,245].

The main drawback of PEI-siRNA polyplexes is the toxicity linked to the high molecular weight of PEI. To lower toxicity, strategies similar to those used for reducing toxicity in PLL have been employed, such as: (i) decreasing the molecular weight to around 25 kDa [240,244]; (ii) reducing the positive Z-potential of the polyplex by partially neutralizing the peripheral amino groups [136,240,244]; (iii) adding hydrophobic residues [246]; (iv) coating the surface of the polyplexes with PEG [238,244,247]; (v) using copolymers [136,244]; (vi) embedding the polyplex in more complex nanosystems [136,139,233,236,241,248].

- CS-siRNA polyplexes. Chitosan (CS) is the most widely used natural polymer for preparing polyplexes that directly deliver siRNA to cells or form part of other nanosystems. It is a linear polysaccharide derived by deacetylation of chitin, composed of repeated units of N-acetyl-D-glucosamine and D-glucosamine linked via β-1,4 bonds. The proportion of these units determines the polymer’s degree of deacetylation (DD) [141,227]. CS is biocompatible, biodegradable, non-toxic, and easily modified [227,249,250]. siRNA can be incorporated into CS through: (i) encapsulation; (ii) adsorption; or (iii) electrostatic interactions [249,251]. The most common method is electrostatic complexation, as the high positive charge of CS facilitates binding with the negatively charged phosphate groups of siRNA [137,227,250].

CS-siRNA polyplexes are stable, their endosomal escape is effective, and they do not cause significant changes in the biological activity of siRNA [141,252,253,254]. The size, shape, and charge of these polyplexes heavily depend on the molecular weight of the CS and the molar ratio between both components (N/P ratio), which then influence transfection efficiency [251,253,255,256].

Despite these qualities, the effectiveness of polyplexes can be hindered by certain physicochemical factors, which include: (i) low stability, especially in those using low molecular weight CS [251,257]; (ii) a tendency to aggregate [257]; (iii) limited cell internalization [250,258]. To improve stability, it is advisable to: (i) use CS with a molecular weight over 10 kDa [249,250,251]; (ii) chemically modify CS [249,250]; (iii) coat the polyplex surface with polymers [137,141,256]; (iv) add CPP or targeting ligands [250,258,259]; (v) incorporate the polyplex into other nanocarrier systems [260,261].

- PBAE-siRNA polyplexes involve polymers prepared by polymerizing diacrylate and amino compounds. These polymers were designed to enhance biodegradability and reduce cytotoxicity compared to PEI and PLL [262,263,264]. They come in diverse structures and shapes, such as linear, spherical, and multi-layered films [231,265]. Their chemical modifications allow for control over size, surface charge, hydrophobicity, degradability, and stimulus responsiveness, influenced by factors like chain length, structure, end groups, solution pH, and N/P ratio [12,231,262,266]. Typically, these polyplexes are serum-stable and readily taken up by cells [12,262]. Inside the cell, they quickly degrade, releasing siRNA and PBAE is hydrolyzed into biocompatible products [12,231]. Their transfection efficiency is influenced by terminal groups, while toxicity relates to hydrophobicity levels and the spacing of amino groups [267]. Adding groups like amines or hydroxyls to the PBAE end chains notably enhances transfection, and the alkyl chain length and end-group hydrophobicity directly impact toxicity [264].

Amphiphilic polymers, also known as polysaccharide peptide conjugates, are utilized for siRNA delivery [268]. These complex molecules are prepared by covalently attaching a polysaccharide, a carbohydrate polymer, to a polypeptide. Various architectures, such as polysaccharide–short cationic peptide and polysaccharide–cationic polypeptide, can be designed for siRNA conjugates. They outperform peptides and polysaccharides alone, being fully biodegradable, offering better biocompatibility than other polymers, and facilitating enhanced endosomal escape due to amino acid residues with proton groups (H+). Additionally, they can recognize cell receptors depending on their amino acid sequence. Although not yet widely used in siRNA delivery, they represent a promising platform, especially since they have demonstrated superior response compared to the well-established PEI polymer.

Other polymeric nanoparticles used to deliver siRNA in cancer treatment include siRNA polyplexes with (i) poly(lactic acid co-glycolic acid) (PLGA) [269]; (ii) α, β-poly(N-2-hydroxyethyl)-D,L-aspartamide (PHEA) [139]; (iii) poly(amidoamine) (PAMAM) [270]; and (iv) Poly(2-dimethylaminoethyl methacrylate) (PDMAEMA) [236], among others.

(b) Lipid Nanosystems

Lipid nanosystems are currently the most commonly used vehicles for siRNA delivery. They naturally enhance cell uptake, protect siRNA from nuclease degradation, and reduce renal clearance. Their key properties, such as size, structure, surface charge, and transfection efficiency, depend on the specific lipids used, allowing their biological functions to be tuned. These nanosystems are biocompatible, biodegradable, less immunogenic than polymeric alternatives, highly effective in delivering siRNA, and are easily and inexpensively produced. They are taken up through various endocytosis pathways. However, a drawback is their tendency to become trapped in the liver [223,271,272,273,274]. They are mainly categorized into two groups: lipid nanosystems (LNPs) and lipoprotein transporters.

LNPs consist of acylglycerides, fatty acids, phospholipids, and steroids, forming mostly spherical structures. Based on their composition and structure, they can be categorized into lipoplexes, liposomes, solid lipid nanoparticles (SLN), nanostructured lipids (NLC), stabilized nucleic acid lipid nanoparticles (SNALP), and lipid-polymer hybrid nanoparticles (see Figure 4 middle panel). Notably, self-nanoemulsifying drug delivery systems (SNEDDS), which are taken orally, and cubosomes, regarded as the third generation of LNPs, are also critical.

Lipoplexes. There are different perspectives on how to define and structure lipoplexes. Many authors refer to all LNPs as lipoplexes, while others consider only liposomes as lipoplexes. In this work, a lipoplex is defined as a supramolecular structure formed by a lipid and siRNA (Figure 4 middle panel). These lipoplexes form spontaneously through electrostatic interactions between the negatively charged siRNA and the positively charged polar heads of the lipid [275,276]. Therefore, lipids with a permanent positive charge (cationic lipids) or zwitterionic lipids that are neutral at physiological pH (7.4) but become positively charged at lower pH (5.5–6.5) due to protonation of their amino groups (ionizable lipids) are used to prepare lipoplexes. Anionic lipids are rarely used for this purpose because they require a cationic mediator, have low cell penetration capacity, and can trigger an immune response [277]. Lipoplexes can also be produced by covalent linkage to the 3′ end of the sense strand of siRNA, but this approach is less common [196].

The electrostatic interaction between the cationic head group of the lipid (either cationic or ionizable) and siRNA results in the formation of thermodynamically stable particles with various morphologies, sizes, stabilities, and interactions with cell membranes. These properties depend on the specific characteristics of the lipid and the preparation conditions [275,278,279]. Cationic lipids used for producing polyplexes include: (i) N-(1-[2,3-dioleoyloxyloxy]-propyl)-N,N,N-trimethylammonium chloride (DOTMA) [272,280,281,282]; (ii) 2,3-dioleoloxy-N-[2-(sperminacarboxamido)ethyl]-N,N-dimethyl-1-propaniminium trifluoroacetate (or chloride) (DOSPA) [276,283,284]; (iii) N,N-dioleoyl-N,N-dimethyl ammonium chloride (DODAC) [272]; (iv) dimethyl-dioctadecyl ammonium bromide (DDAB) [285,286]; (v) N-(N′, N′-dimethylamino ethane)-carbamoyl cholesterol (DC-cholesterol) [283]; (vi) 1,2-dioleoyl-3-trimethylammonium propane sulfate (DOTAP) [195,285,287]. These lipids consist of three parts: (i) a polar head containing one or more amine groups, which can be primary, secondary, tertiary, or quaternary; (ii) two hydrophobic chains, which may be saturated or unsaturated; and (iii) a connecting group linking parts (i) and (ii). The amine groups are responsible for complexing with siRNA, the hydrophobic chains aid in packaging, and the connector enhances the chemical and enzymatic stability of the nanosystem [275,281,285].

The transfection ability of lipoplexes is high. Their overall positive charge helps them interact electrostatically with the negatively charged lipids of cell membranes, allowing entry into the cell through endocytosis [198,288]. However, cationic lipoplexes are cleared from the plasma very quickly and tend to form aggregates and micelles, which are absorbed by macrophages in the lungs, liver, and spleen. In contrast, their uptake in tumors and inflammation sites is lower, leading to reduced silencing efficiency [198,272,289]. To address these issues, additional polymers and other components are incorporated to create liposomes and more advanced lipid nanosystems [290].

Ionizable pH-dependent lipids are preferable to permanent cationic lipids. Lipoplexes with ionizable lipids are prepared at low pH to ensure electrostatic interaction with siRNA [281,289]. Because they are neutral (pKa < 7) at physiological pH, their interaction with blood cells is less than that of cationic lipids [237,272,278]. Once inside the cell, they protonate again, interact with the negatively charged endosomal membrane, and destabilize it, allowing the siRNA to be released easily into the cytosol. Ionizable lipids used for preparing siRNA lipoplexes include: (i) dioleoyl-phosphatidyl ethanolamine (DOPE) [273,290,291,292]; (ii) 1,2-dioleoyl-3-dimethylammonium propane (DODAP) [272,293]; and (iii) 1,2-dilinoleyloxy-3-dimethylaminopropane (DLin-DMA) and its derivatives [16,196,271,272,273,294]. All of these are used to formulate various lipid nanosystems.

The silencing ability of lipoplexes depends on the pKa of the amino group, the unsaturation of the acyl chains, and the structure of the connectors [272,281,289]. Ionizable lipid-like molecules, called lipidoids, are now being synthesized, with their structure designed through specific combinations of the three lipid components to enhance the in vivo efficacy and safety of LPNs. Among the most commonly used are 98N12-5, C12-200, OF-02, L3-19 [16,272,295,296]. Patrisiran was the first siRNA formulation approved for clinical use [237]. Although it was not developed for cancer therapy, it is a carrier nanosystem based on ionizable lipids. The advantages of ionizable lipids as targeted delivery systems for siRNA were recently reviewed [237].

Liposomes. Liposomes are the most commonly used LNPs for siRNA transport and delivery. They are spherical vesicular structures composed of one or more phospholipid bilayers (natural or synthetic) that self-assemble around a central aqueous core when dispersed in water (Figure 4 middle panel). Hydrophobic interactions and Van der Waals forces stabilize them. The phospholipid bilayers are separated by aqueous compartments. This structure enables the encapsulation of hydrophilic molecules in aqueous compartments, while molecules with different characteristics can be trapped or attached to the lipid bilayer. Molecules with intermediate log P are retained in both the aqueous compartments and the lipid bilayer. These features enable liposomes to carry multiple types of molecules simultaneously, making them helpful in developing theranostic and multimodal therapy systems. The physicochemical and biological characteristics of liposomes—such as structure, size, lipid composition, surface charge, bilayer fluidity, and interaction with cell membranes—depend on their component combinations and production methods [297,298,299,300]. For example, the number of bilayers influences size and encapsulation efficiency [271,289,300,301]. For medical applications, single bilayer (unilamellar) liposomes with sizes of 50–150 nm are preferred [300]. 

Key components of siRNA-carrying liposomes include (i) phospholipids that form lipoplexes with the siRNA and promote its endosomal escape; (ii) hydrophobic lipids that provide stability to the liposome; (iii) polymeric molecules that coat the liposome to enhance pharmacokinetics; and often (iv) ligands targeting cell receptors [271,276,289,299,300]. The hydrophilic head of the phospholipids (positive, negative, or zwitterionic) and their hydrophobic tail define the amphipathic nature of the bilayer and the surface charge of the liposome [276,300]. 

The most commonly used natural phospholipids are phosphatidic acid (PA), phosphatidyl ethanolamine (PEA), phosphatidyl glycerol (PG), phosphatidyl serine (PS), phosphatidyl choline (PC), and phosphatidyl inositol (PI) [300,302]. These phospholipids are modified to obtain synthetic derivatives with improved characteristics, such as DOPE, DOTAP, DOTMA, dilauryl phosphatidyl choline (DLPC), 1,2-dimyristoyl-sn-glycero-3-phosphocholine (DMPC), 1,2-dipalmitoyl-sn-glycero-e-phosphatidylcholine (DPPC), 1,2-distearoyl-sn-glycero-3-phosphocholine (DSPC), dilauryl phosphatidyl ethanolamine (DLPE), dilauryl phosphatidyl glycerol (DLPG), dioleoyl phosphatidyl serine (DOPS), 1,2-dioleoyl-sn-glycero-3-phosphatidylcholine (DOPC), among others [272,278,283].

To improve bilayer properties and in vivo stability, hydrophobic lipids (co-lipids) are added. These lipids, usually non-cationic, include sterols, phospholipids, and glycerolipids. The most used is cholesterol and its derivatives [283,291,300], although DSPC, DOPE, and DOPC are also used and contribute to fusion with cell membranes [275,283,299,300,303]. Cholesterol fills the empty spaces between phospholipid molecules, inserts with the -OH groups facing the aqueous phase, and the aliphatic chain aligned parallel to the acyl chains of the hospholipids. In this way, it contributes to the rigidity of the bilayer, reduces the permeability of hydrophilic compounds through the bilayer and the tendency of liposomes to aggregate while increasing the absolute Z-potential, the blood lifetime, the permeability of hydrophobic compounds, and promotes interaction with cell membranes [272,276,291,299,300,304]. 

To increase the lifetime in blood, decrease MPS recognition and toxicity, it is common practice to coat liposomes with different molecules. The most used is PEG, natural or modified [272,273,305,306]. In addition to improving pharmacokinetics, PEG allows antibodies, antibody fragments, peptides, aptamers, radionuclides, and ligands to be conjugated to the liposome surface, enabling the targeting of specific cell receptors [307,308,309]. As natural PEG has the disadvantage of negatively influencing cell uptake and endosomal escape of siRNA, and may cause unwanted toxicity, derivatives have been developed to improve both properties and increase transfection capacity [305,306].

The method of liposome preparation influences their physicochemical and biological properties [271,289,299,302,308]. Depending on the method, liposomes with different structural and surface properties can be obtained, which interact with cells by multiple mechanisms [299,300,306,310]. 

The siRNA can be inserted into the aqueous core (encapsulation) or into the lipid bilayer of liposomes. The site of insertion influences subsequent biodistribution [298,305,311]. Encapsulation is preferred as it ensures greater stability in vivo. Encapsulation can be carried out during the preparation [298,312,313,314,315] or after liposome preparation [303,310,316,317]. If the encapsulation is done during the preparation (passive encapsulation), naked siRNA is used [297] or in the form of positively charged complexes [274,293]. Suppose the encapsulation is performed after the liposome has been prepared (active encapsulation). In that case, the naked siRNA is not suitable because if the liposome contains PEG, the addition of the siRNA may form a very large nanosystem or the siRNA may adhere to the liposome surface and be released as soon as it enters the bloodstream [297,298]. 

The insertion of siRNA into the lipid bilayer depends on the phospholipids, the preparation conditions, and the N/P ratio [271,289,298]. The simplest variant of incorporation into single-layer liposomes is to pre-conjugate the siRNA to hydrophobic molecules such as cholesterol and fatty acids [289,306,318,319]. Binding by adhesion to the surface can be done either with naked siRNA (cationic liposomes) or in the form of conjugates [298].

Lipofectamine 2000 is the most used commercial liposome for siRNA transfection, especially in vitro. It consists of a cationic liposome made from a 3:1 mix of DOSPA and DOPE, enabling high-efficiency and reproducible transfection across various cell lines [272]. However, a limitation of liposomes is their instability during storage, which has prompted the development of more stable nanosystems.

Solid lipid nanoparticles (SLN) and nanostructured lipids (LNC) represent a more advanced generation of lipid-based carriers than liposomes, with a stiffer morphology, higher physical stability, and greater loading efficiency [272,274,276]. They are easy to produce, stable during freeze-drying, do not require organic solvents for preparation, and can release encapsulated siRNA in a controlled manner. SLNs consist of a solid lipid core surrounded by a layer of non-ionic surfactant [274,314,320]. They may or may not be coated with additional materials [321]. Typical lipids used in SLNs include fatty acids, fatty alcohols, glycerides, and waxes with a melting point above 40 °C [274,276]. Surfactants are employed to reduce interfacial tension between the lipid and aqueous phases, enhance formulation stability, and influence the release kinetics of the encapsulated siRNA [321,322]. Many combinations of lipids and surfactants are available to create SLNs with different physicochemical and biological properties, which can incorporate siRNA in either hydrophilic or lipophilic forms. To load siRNA into the SLNs’ lipophilic core, it must first be conjugated, preferably as lipoplexes [323].

LNCs are modified SLNs (Figure 4 middle panel). They are considered as some of the most advanced lipid carriers for siRNA delivery [272,314]. They consist of a mixture of solid and liquid lipids, to which surfactants are also added [274,314,324]. They exhibit higher loading efficiency and stability than SLNs, with similar toxicity levels. Their capacity to internalize tumor tissue is also high [314]. Both SLNs and LNCs are produced using several methods summarized in different publications [324], although all methods involve incorporating the siRNA into melted or liquid lipids before mixing with the surfactant solution.

Stabilized nucleic acid lipid particles (SNALP) can be considered a specific type of liposomes (Figure 4, middle panel) [325]. They have a size of approximately 100 nm and a neutral charge. They consist of four main components: (i) a lipid that can be cationic or ionizable to form the lipid-siRNA lipoplex that is encapsulated in the nucleus; (ii) one or more ionizable lipids forming the membrane and contribute to the siRNA endosomal escape and cytosolic release; (iii) one or more high-transition-temperature ancillary lipids that stabilize the lipid layer; (iv) a PEG lipid derivative to enhance pharmacokinetics and target cell surface molecules [326,327,328]. Cationic and ionizable lipids are the ones previously mentioned (see lipoplexes). Generally, auxiliary lipids such as cholesterol, DSPC, and DSPE are used [16,321,326,327].

The most common PEG derivatives are diacylglycerols like PEG-DSG and PEG-DMG [327,329]. Unlike liposomes, SNALPs have a micelle-like structure. In these micelles, the hydrophobic core contains the lipoplex, the bilayer is composed of the ionizable neutral lipid, cholesterol, and auxiliary phospholipids, and the surface features the PEG lipid conjugate, which may or may not have targeted molecules attached [328]. Several methods are used to prepare SNALPs, but the most common involves microfluidic mixing of lipids and siRNA in ethanol followed by dialysis [297,327]. After mixing and prior to dialysis, connectors are added.

Lipid-polymer hybrid nanoparticles (LPHNPs) combine the benefits of lipid and polymeric nanoparticles. Their pharmacokinetic properties, biocompatibility, bioavailability, shelf stability, and ability for controlled release are superior to those of lipid or polymeric nanoparticles alone, while they exhibit lower immunogenicity and toxicity [183,330,331,332]. Their main components include siRNA, a lipid, and a biodegradable polymer. These nanoparticles are divided into three sections: (i) the polymeric core, where siRNA is efficiently encapsulated as a polyplex; (ii) an inner lipid monolayer formed by a cationic lipid that surrounds and protects the core, promotes biocompatibility, and enables controlled siRNA release; (iii) an outer layer composed of a hydrophilic lipid-PEG (or lipid-PVP) conjugate that enhances stability and pharmacokinetics and allows attachment of molecules targeting specific cell receptors (Figure 4 middle panel) [183,331]. Due to their multimodal loading capacity, they are ideal for designing theranostic systems [333,334]. Additionally, siRNA can be incorporated into the lipid bilayer through covalent bonding [335].

The polymers used to prepare the LPHNPs core include those described earlier. Commonly used ones are PEI, PLGA, polycaprolactone (PCL), poly-lactic acid (PLA), poly-hydroxyethyl methacrylate (PHEMA), PBAE, and CS [183,242,331,333,336,337,338,339,340]. Depending on the polymer used to form the core polyplex, the inner lipid monolayer may consist of cationic, anionic, or neutral lipids. If the core is formed with a charged polyplex, then the inner layer lipids are oppositely charged. If the polyplex is hydrophobic, then the interaction with the lipids occurs through the hydrophobic tails of the lipids [341]. 

The list of lipids used to form the inner and outer lipid layers is extensive. Among the most common are myristic acid, phosphatidylcholine, cholesterol, stearic acid, 1,2-dipalmitoyl-sn-glycero-3-phosphocholine, DOTAP, DSPG, DOPC, DSPE, and glycerylmonooleate (GMO) [330,338,342,343,344]. The variety of lipid/polymer combinations results in LPHNPs with diverse physicochemical, structural, and functional properties [335,340,345]. A table listing some of these combinations can be found in [346].

Although there are different methods of preparing LPHNP, they are all based on mixing its components in one or more stages [264,274,331,335,340]. There are also hybrid nanoparticles that are prepared with inorganic/organic components.

Self-emulsifying drug delivery systems (SNEDDS) are lipid-based vehicles suitable for oral siRNA transport. They are thin oil-in-water (o/w) nanoemulsions smaller than 100 nm, consisting of an isotropic and stable mix of lipids, surfactants, cosurfactants, and cosolvents, in which active substances are dissolved or suspended [347,348]. These systems undergo self-transformation in situ when their oily phase contacts the aqueous environment of the intestinal tract (GIT). GIT motility creates agitation, allowing spontaneous formation [347,349]. They protect against enzymatic hydrolysis, have a fast onset of action, higher loading efficiency compared to other lipid systems, and can be formulated in liquid or solid forms. They are easy to manufacture and scale up. Additionally, they can incorporate both hydrophilic and lipophilic molecules and are therefore an alternative for siRNA delivery [348,349].

Other lipid siRNA transport systems, including cubosomes or cuboplexes, are at earlier stages of pharmaceutical development [350,351]. Cubosomes are LNPs with an internal bicontinuous cubic symmetry and have higher transfection efficiency than liposomes, probably because of their different mechanisms of interaction with cells [352]. They are considered the third generation of LNPs.

(c) Lipoprotein-Based Nanosystems

Lipoproteins are complex assemblies of lipids and proteins with varying sizes and densities, present in blood serum. They are crucial for transporting cholesterol, triglycerides, and other lipids, and also participate in processes such as coagulation, tissue repair, and immune responses [353]. Their hydrophobic core, consisting of triglycerides and non-polar cholesterol esters, is covered by a monolayer of phospholipids and free cholesterol, into which specialized proteins called apolipoproteins are intercalated to contribute to the stability and facilitate recognition by cell receptors [308,354]. Free cholesterol, located between the alkyl chains of phospholipids, helps maintain the outer layer’s rigidity [355]. These lipoproteins are classified by size, composition, and density into: (i) chylomicrons (CM, >100 nm), (ii) very low-density lipoproteins (VLDL, 30–80 nm), (iii) intermediate-density lipoproteins (IDL, 30–50 nm), (iv) low-density lipoproteins (LDL, ~25–35 nm), and (v) high-density lipoproteins (HDL, 5–12 nm). In living organisms, they can convert from one type to another based on the current lipid-to-protein ratios [353,354].

LDL and HDL (Figure 4 bottom panel) are employed as drug carrier systems due to: (i) they are not recognized by the MPS; (ii) they have long blood circulation times; (iii) they are non-immunogenic; and (iv) they can transport hydrophobic, hydrophilic, and amphiphilic molecules without significantly altering the structure or biological properties of the transported molecule. They can be used in native forms (isolated from plasma) or in synthetic forms. The native form is less practical because isolation, purification, and quantification involve lengthy and costly procedures that are not very scalable and are hard to standardize [353,354,355]. It is preferable to synthesize them from their natural or synthetic components (reconstituted, rLDL, and rHDL). Reconstituted lipoproteins, where the apolipoproteins are replaced by mimetic peptides, are called mimetics (mLDL and mHDL) [354,356,357]. These synthetic lipoproteins offer the advantage that their component ratios can be adjusted to create nanoparticles with specific physico-chemical properties, without compromising the biological functions of native lipoproteins. They are easy to produce and well tolerated by patients [308,354,358]. Compared to liposomes, they are smaller and can directly interact with targeted cell receptors.

Synthetic LDL and HDL NPs are typically spherical, though they can also be discoidal [357,359]. They can attach biomolecules and ligands to their surfaces, allowing them to target their own cell receptors as well as other receptors of interest on the cell membrane. They are internalized in cells by both passive and active transport [357,360]. Their multimodal loading capacity supports the development of theranostic systems [360,361,362] and multimodal therapy systems [363,364]. They break down into recyclable units of cholesterol, fatty acids, and amino acids and do not require PEG coating to extend their blood circulation times [353,355]. These advantages have contributed to their use in diagnosing and treating various types of tumors.

LDL-siRNA nanosystems. LDL is the primary cholesterol transporter to different extrahepatic tissues and plays a crucial role in their homeostasis and steroid synthesis. Native LDL is a diverse group of particles that vary in size, density, and composition (Figure 4 bottom panel). Apo B-100 is embedded in the amphiphilic monolayer of phospholipids and free cholesterol [353,355,365]. It is among the largest proteins known (>500 kDa, 4536 amino acids). Apo B-100 encircles the LDL surface like a belt, contributing to its stability and mediating receptor (LDLR) binding [355,365]. LDL and HDL compositions are detailed in Table 3.

The tissue with the highest expression of LDLR is the liver, but the adrenal glands, reproductive organs, skeletal muscle, lymphocytes, kidneys, and intestines also express these receptors, although in smaller amounts [353,355]. Some tumors overexpress LDLRs because they need lipids for growth and proliferation [355,359,365,366,367,368,369,370,371]. Cholesterol is also a precursor to steroid hormones, which are important in the development of breast and prostate cancer [367,372]. These facts have motivated the use of LDL for cancer diagnosis and therapy.

Although HDL is more commonly used than LDL for siRNA delivery, cholesterol-siRNA conjugates can be effectively loaded into LDL [355,368,373]. The main pathway for internalizing cholesterol-siRNA conjugates via LDL involves ApoB-100 binding to its receptors (LDLR) [353,368,373]. After endocytosis, LDL merges with lysosomes, where it is hydrolyzed, releasing its contents into the cytosol. LDLR is then recycled back to the cell surface, while its components are degraded, and the siRNA is freed from cholesterol to bind to RISC [353]. 

Various methods have been developed for rLDL preparation [355,365], which utilize natural or synthetic lipids to which Apo-B 100 is added [353]. Some researchers also include Apo E [374,375]. A challenge with ApoB-100 is that it is difficult to isolate and tends to aggregate, making mimetic peptides a more practical option [375].

Naked siRNA is not naturally incorporated into LDL, so it must be conjugated with other molecules. For example, lipophilic conjugates such as cholesterol-siRNA and fatty acids-siRNA are used [368,370,373,376]. Amphiphilic conjugates designed to integrate into the phospholipid layer are also employed. The amino acids of ApoB100 allow covalent attachment to various molecules, including peptides, proteins, antibodies, antibody fragments, and aptamers.

Lipophilic cholesterol-siRNA and fatty acid-siRNA conjugates are incorporated into the LDL core during nanosystem preparation through exchange with core lipids [355,362,373]. Alternatively, some researchers prefer to perform this exchange after preparing rLDL [368,374]. These lipophilic conjugates, encapsulated within the rLDL core, are mainly absorbed by the liver, with limited uptake in other LDLR-expressing organs [368,373], although brain uptake has also been observed [374].

siRNA conjugates are incorporated into rLDL phospholipids through both covalent binding via phosphodiester bonds and non-covalent interactions by embedding conjugate hydrophobic tails, which orient hydrophilic heads toward the aqueous environment. In this way, the conjugate is inserted via Van der Waals forces, hydrogen bonds, and ionic interactions [355,362]. The balance and strength of Van der Waals forces between the inserted molecule and phospholipids, along with the hydrogen bonds and ionic interactions, determine the efficiency and stability [355,362]. However, this method has the drawback that some molecules tend to escape from rLDL.

For siRNA binding to Apo B100, specific residues such as lysine, arginine, tyrosine, and cysteine are utilized to attach molecules that target cellular receptors other than LDLR [355,362,377]. For example, FA can be attached to ApoB-100 by pre-modifying lysine residues [377]. Although this reduces ApoB-100’s ability to bind LDLR, it allows rLDL to bind to FR, which is expressed in various cancers. Alternatively, surface modification of rLDL with cationic polymers can create electrostatic complexes with siRNA, providing attaching to rLDL [365]. 

HDL-siRNA nanosystems use HDL, the smallest and densest lipoprotein, which is crucial for reverse cholesterol transport. Similar to LDL, HDL contains various NP subpopulations that affect its functions in vivo [372,378]. It differs from LDL in its component ratios and protein composition (see Table 3). Its main protein, Apo A-1, accounts for about 70%, influencing HDL’s size and appearance, allowing its binding to SR-B1 receptors, which are often overexpressed in tumor cells [358,372,379,380]. Additionally, Apo A-1 has anti-tumor effects [381].

Physicochemical properties of rHDL are similar to those of native HDL [361,382]. When rHDL interacts with SR-B1, a hydrophobic structure forms. This enables the direct release of transported molecules into the cytosol [383,384]. This interaction is especially beneficial when SR-B1 is overexpressed, as it enables selective delivery of therapeutic molecules [385].

rHDL prevents siRNA endolysosomal degradation and offers high transfection efficiency [373,380,386,387]. Similar to rLDL, siRNA can be incorporated into both the core and the outer layer. To incorporate siRNA into the rHDL core, hydrophobic conjugates with an octanol:water partition coefficient, expressed as Log P > 3, are required. Conjugates with Log P > 1 can also be used. Encapsulation can occur during preparation [361,379,387] or after the rHDL is formed [374,388,389]. In the first approach, the molecule is exchanged or previously bound to one of the core components. In the second, the desired agent is introduced after the rHDL has been prepared.

Cholesterol is commonly used as a hydrophobic agent to bind siRNA to rHDL [202,374,390,391,392]. Oligosine [379], fatty acids [202,373], α-tocopherol-siRNA [388] are also used. These conjugates are encapsulated in the core or inserted into the bilayer depending on their physicochemical properties and preparation method. Some authors have coupled siRNA to the rHDL surface through electrostatic interactions. In this case, cationic polymers or lipids are bound to rHDL surface to form polyplexes or lipoplexes with siRNA [356,379,389]. Covalent binding by hybridization has also been used [356]. The siRNA chemical structure, together with the preparation method of rHDL/siRNA complexes, critically affects NP–receptor interactions and transfection efficiency [393]. These variables are considered required.

Similar to rLDL, the insertion of molecules into the rHDL surface occurs through: (i) covalent bonding to the head groups of phospholipids [308,360,362] or to the lysine, arginine, tyrosine, or cysteine residues of Apo A-1 [362,394,395], and (ii) non-covalent intercalation of conjugates with membrane components via Van der Waals forces, hydrogen bonds, or ionic interactions [308,357,362,396].

An advantage of rHDL is that it can simultaneously transport several molecules, which enables the design of theranostic systems and multimodal therapy [361,397,398]. However, it has the disadvantage that it is taken up by the liver, kidneys, stomach, steroidogenic tissues, and smooth muscle [379,380], which could cause unwanted off-target effects.

(d) Cell-Derived Nanosystems

Organic nanosystems derived from cells, called extracellular vesicles, are in the pharmaceutical development stages. These nanosystems are small vesicles (30–200 nm) secreted by various types of cells, including tumor cells. They play different roles in cell communication, the immune system, tissue repair, and facilitate the transport of bioactive molecules [399,400]. They have numerous advantages because they easily cross biological membranes, have long circulation times in the blood, and are non-toxic [400]. There are different subtypes of extracellular microvesicles, but the most studied as siRNA transporters are exosomes [320,399,401,402]. Its main drawback is the low siRNA loading capacity and high hepatic uptake [402]. Efforts are currently focused on developing strategies to overcome these challenges.

(ii) Inorganic and Hybrid Nanocarriers

Over the past twenty years, inorganic nanosystems (metallic and non-metallic) have been developed for siRNA delivery, offering better mechanical properties than organic nanoparticles, sufficient bioavailability, low toxicity, and high stability in serum, among other beneficial qualities. The surface of these inorganic nanoparticles, which vary in morphology and physicochemical characteristics, can be easily modified through covalent conjugation with organic ligands or amphiphilic polymers, electrostatic interactions, or coating with lipid systems [384]. They are useful in designing theranostic systems and multimodal therapies [278,403,404]. These are also called multifunctional nanoparticles and hybrid nanoparticles [384,405]. Their great diversity enables various strategies for coupling siRNA through both covalent and non-covalent bonds [384,404]. The most commonly used include: (i) gold nanoparticles; (ii) magnetic nanoparticles; (iii) inorganic semiconductor crystals (quantum dots); (iv) carbon-based nanoparticles; and (v) mesoporous silica nanoparticles. This category also includes calcium phosphate-siRNA complexes encapsulated in lipid systems.

- Au nanoparticles. These are colloidal suspensions of Au with unique chemical and physicochemical properties that have been used in biomedicine since ancient times. They absorb light and X-rays, and their absorption maxima can be adjusted during synthesis. They can disperse and convert absorbed light into heat, enhance the Raman spectra of molecules near their surface, produce fluorescence, and carry different molecules with high loading capacity. They are bioinert, low in toxicity, and easy to obtain [406,407,408]. They easily conjugate to biomolecules due to their high affinity for thiols, disulfides, and amine groups [384,409,410]. They efficiently transfect siRNA without needing additional transfection agents.

siRNA binds to AuNPs through covalent bonding or electrostatic interactions. For covalent attachment, AuNPs are functionalized with amine, cysteamine, or azide groups [407,411,412,413], and then the modified siRNA is linked via the bioconjugation reactions already described. Alternatively, siRNA can be directly attached to the surface of AuNPs after modification with thiol groups, forming an Au-S bond [404,414]. This method also allows hybridization-based binding [415]. Some researchers attach PEG functionalized with amine groups or thiols to the AuNP and then connect the siRNA to the PEG using a linker [414]. Others conjugate siRNA to PEG first and then attach the siRNA-PEG complex to the AuNP [412].

For AuNP-siRNA binding through electrostatic interactions, the AuNP is positively charged before adding the siRNA, which can be naked or conjugated to a linker. Cationic polymers and peptides are used to positively charge the AuNP. One of the most common is PEI, due to its dual role as a reducing agent and stabilizer of Au+ ions, as well as its strong electrostatic interaction with siRNA [416,417,418]. However, its toxicity is a drawback, which is why some researchers prefer CS [419,420,421]. Among cationic peptides, protamine is the most used, though TAT and RGD have also been employed [410,422]. Electrostatic bonding enables layer-by-layer assembly [417,420].

Since the physicochemical properties of AuNPs depend on their shape and size, they are prepared in various forms—such as spheres, rods, stars, cubes, and plates—to suit different applications. This variety broadens the range of therapeutic options [409,423]. Spherical AuNPs are used to produce so-called spherical nucleic acids (SNAs).

AuNP/siRNA coated with cationic carboxylane dendrimers has demonstrated its therapeutic efficacy for silencing antiapoptotic proteins of the BCL family, which are overexpressed in different tumors [424]. Other systems, where AuNP/siRNA are coated with PEG-acrylate and folate monomers, have been used to promote tumor vessel normalization, enhance immunotherapy, and inhibit metastasis formation [425].

- Magnetic nanoparticles are used to develop NMR theranostic systems and for gene silencing. They consist of materials containing ferromagnetic, paramagnetic, diamagnetic, antiferromagnetic, and ferrimagnetic elements, with sizes ranging from 10 to 20 nm. These particles exhibit different responses to external magnetic fields [426,427]. Superparamagnetic iron oxide nanoparticles (Fe_3_O_4_ and γ-Fe_2_O_3_), known as SPIONs, are the most commonly used in medicine due to their versatility, biocompatibility, strong magnetization, and low production costs [428,429,430].

Like other inorganic systems, magnetic NPs are functionalized with different molecules, and siRNA is assembled using both covalent bonds and electrostatic interactions. This results in systems with diverse morphology, size, surface charge, pharmacokinetics, and magnetic properties [405,427,429,431,432]. The methods employed for siRNA coupling are similar to those used for AuNPs.

Among the most commonly used molecules to attach siRNA to magnetic NPs are cationic polymers, with PEI standing out [405,428]. Cationic peptides have also been utilized [433], along with slightly anionic polymers such as PEG and its derivatives [429,430,434], polysaccharides [427], and combinations of molecules [435,436]. The concurrent binding of siRNA and molecules targeting specific cellular receptors enhances the efficiency of these nanosystems [437].

An advantage of magnetic NPs is that siRNA can be concentrated in a given tissue by applying an external magnetic field. The method, called magnetofection, depends on the interaction of many variables including NP composition, the applied magnetic field, the strength of the siRNA-NP bond, the route of administration, and the location and depth of the target tissue [426,438]. It has been shown, however, that it offers the possibility of internalizing siRNA in difficult-to-transfect cells such as neuronal cells [437,438].

- Inorganic semiconductor crystals, known as quantum dots (QDs). They are nanocrystals ranging from 2 to 20 nm that are created from binary combinations such as CdSe, CdTe, CdS, ZnS, ZnHgSe, PbS, CdHgTe, and CdxPb1-xTe alloys. Their optical properties vary with changes in composition, size, and shape [439]. These QDs offer several advantages: (i) tunable emission; (ii) high fluorescence quantum yield; (iii) resistance to photobleaching; (iv) a high surface-to-volume ratio; and (v) ease of functionalization, which facilitates the transport of molecules of diverse types [440]).

The physicochemical properties of QDs depend on the method of production, which can be physical, chemical or biological [440,441]. However, QDs offer the possibility of simultaneously coupling molecules of different nature and obtaining high contrast images, which is useful for theranostic devices [442,443,444,445]. They have the advantage of being easily integrated into LNP.

siRNA binds to QDs through both covalent and non-covalent interactions. The QD surface is functionalized with molecules containing terminal groups such as carboxyl, amine, thiol, hydrazine, or diol [384,441,446]. siRNA is then attached via established reactions, including (i) disulfide bridges, (ii) carbodiimide-amine coupling, (iii) thiol-maleimide reaction, and (iv) azide-alkyne click chemistry. Non-covalent coupling occurs through affinity and electrostatic interactions. For affinity coupling, streptavidin is attached to the QD followed by biotinylated siRNA [441]. Electrostatic coupling involves cationic molecules first attaching to the QD, then linking siRNA. Common functionalization molecules include PEG and derivatives [446,447,448], PEI [447,449,450], peptides [451], saccharides [443], and amino acids [442]. To enhance biocompatibility and transfection efficiency, some researchers use combinations of these molecules [445,447,448] or more advanced nanosystems [452,453]. Recently, carbon QDs (carbon dots, CD) have emerged as an alternative.

- Carbon-based nanoparticles leverage the chemical properties of carbon to create carrier NPs with sizes akin to many biological structures. Hybridization of sp^2^ carbon enables the production of various graphitic materials—such as nanodiamonds (3D), graphene (2D), nanotubes (1D), fullerenes, and quantum dots (0D)—which are used to fabricate NPs with diverse structures. These materials offer exceptional mechanical strength, high stability, a large surface area-to-volume ratio, unique optical features, excellent thermal and caloric conductivity, biocompatibility, ease of functionalization, and antibacterial properties [454,455]. For siRNA coupling, the primary options include: (i) carbon nanotubes, (ii) graphene oxide nanosheets, (iii) fullerenes, and (iv) carbon quantum dots.

Carbon nanotubes (CNTs). These are elongated tubular nanostructures, measuring 50–100 nm in length, made from graphite sheets derived from carbon atoms arranged in a hexagonal pattern. They exhibit high biocompatibility, excellent electrical and thermal conductivity, and are straightforward to synthesize and functionalize [455,456]. Their optical properties support combined applications in gene and photothermal therapy. CNTs can be produced as either single-walled (SWCNT) or multi-walled (MWCNT) varieties [428]. 

SWCNTs are primarily employed for siRNA delivery, with their surfaces functionalized through covalent bonding, hydrophobic interactions, or van der Waals forces [428,455,457,458,459]. The specific molecule used for CNT functionalization determines whether siRNA is attached via covalent or electrostatic bonds [457,458,459,460]. Amphiphilic PEG derivatives are commonly utilized because they also facilitate the attachment of various biomolecules for receptor recognition [458,461]. The techniques for siRNA coupling have already been detailed.

CNTs have a high loading capacity, and their tubular structure enables them to easily penetrate the plasma membrane, releasing siRNA into the cytosol through a mechanism that does not rely on endocytosis [455,459]. An example of effective CNT/siRNA application is the system developed by Siu et al. [458], who use SWCNTs covalently bonded with PEI-modified lipids, allowing siRNA to bind via electrostatic interactions. However, these systems have been shown to potentially cause cytotoxicity [461]. 

Graphene oxide nanoparticles (GON). These are nanosheets derived from graphene or graphite, with atoms in a hexagonal sp^2^-hybridized configuration. They also contain domains of sp^3^-hybridized carbon atoms [338]. GONs are produced through various methods, with the most common being chemical or thermal exfoliation of graphite oxide, followed by dispersion in water to form stable colloids [462]. Their physicochemical properties are unique, with hydrophilicity and biocompatibility surpassing those of graphene. Additionally, they exhibit high specific surface area, thermal and electrical conductivity, and optical transmittance [338,455,456], enabling the development of multimodal therapy and theranostic systems [445,463,464,465]. The surface of GONs can be easily functionalized to attach siRNA, as hydroxyl, carboxyl, epoxy, and carbonyl groups are highly accessible. Functionalization occurs via both covalent and non-covalent bonding [455,465,466,467,468,469]. Covalent bonding involves various reactions, with nucleophilic substitution of epoxy and carboxyl groups by molecules containing primary amines being one of the most common methods [466,468,469]. Non-covalent attachment primarily occurs through electrostatic interactions using positively charged polymers and peptides [468,470]. The process of coupling siRNA to the functionalized GONs follows the methods previously described.

GONs offer lower toxicity and higher loading capacity than other inorganic nanocarriers, and they can be integrated into various nanosystems to prepare hybrid nanoparticles [468].

Fullerenes. They are three-dimensional, mostly spherical structures composed of large, complex, and highly durable carbon molecules arranged in an alternating pattern of hexagons and pentagons. The most common fullerene is C60, formed by 60 carbon atoms connected by single bonds (12 pentagons) and double bonds (20 hexagons). Their size and non-polar nature enable them to easily penetrate cell membranes [456,471], making them highly suitable for drug delivery. Fullerenes can also be functionalized with molecules of different nature [341,471,472,473]. Additionally, their photoelectrochemical properties make them excellent candidates for photodynamic therapy (PDT) [341,456]. siRNA primarily binds to fullerenes through electrostatic interactions, which requires pre-cationizing the fullerene with molecules containing amine groups [341,473,474,475].

Carbon quantum dots (CDs). These are nanoparticles less than 10 nm in size, exhibiting optoelectronic properties similar to traditional quantum dots while offering greater biocompatibility and lower toxicity [445,469,476]. They are ideal for siRNA delivery. CDs consist of a carbon skeleton with sp^2^/sp^3^ hybrid [477,478]. Their solubility and biocompatibility surpass those of CNTs, GONs, and fullerenes, making them better suited for in-vivo applications [477]. They are produced through diverse low-cost methods, either by top-down techniques that break down larger NPs or by bottom [476,478,479]. Depending on the core structure—crystalline or amorphous—and surface functional groups, they are categorized into four groups. Graphene quantum dots (GQDs), derived from graphene or graphite, have a crystalline structure composed of one or more graphene sheets (up to 10), connected by chemical groups at the edges, and are rich in hydroxyl, carboxyl, carbonyl, and epoxy groups like GONs [469,480]. Carbon quantum dots (CQDs) are produced from small organic molecules or CNTs, feature a crystalline core, and are always spherical [480]. Carbon nanodots (CNDs), obtained from graphite, have an amorphous core and spherical shape, with a high degree of carbonization [478,480]. Carbonized polymer dots (CPDs) are hybrid nanoparticles formed by incomplete carbonization of polymers, with attached functional groups [480].

Like other carbon NPs, CDs are functionalized with groups such as amine, carboxyl, and hydroxyl to enable covalent and non-covalent linking of molecules. These molecules then bind to siRNA, primarily through electrostatic interactions [481,482,483]. The most commonly used molecules have already been described. Their choice for CD functionalization aimed at siRNA binding depends on the intended application—whether theranostic, multimodal, or combined with gene silencing [482,484].

- *Mesoporous silica* nanoparticles (MSNP). These are silica nanoparticles with various morphologies and numerous pores. Their structure, shape, nanoparticle size (50–200 nm), and pore size (2–50 nm) can vary depending on the production method [485,486,487]. They are highly stable under physiological conditions, biocompatible, biodegradable, low in toxicity, and considered safe for in vivo applications by the FDA [488,489]. Adjustable pore sizes enable the loading of small molecules into small nanoparticles [486]. Surface silanol groups (Si-OH) facilitate functionalization with amines, thiols, chlorides, phosphates, carboxyl groups, and others, allowing the attachment of a variety of molecules [488,489,490,491]. This versatility supports the development of controlled release systems [486], targeted therapy [489,490], stimuli-responsive systems [492,493], theranostics [494,495], and multimodal therapy [496,497,498]. They can be either conventional (MSN) or hollow (HMS), with different synthesis methods utilized depending on the specific application, as summarized in several publications [487,499].

siRNA can be incorporated into MSNPs either during their synthesis or afterward, with the latter being more common [81,486]. This can happen inside the pores or on the surface of the MSNPs. For pore incorporation, the MSNPs need to have large pores to allow siRNA, conjugated with various molecules, to enter [489]. However, surface coupling is generally preferred and can be achieved either covalently or via electrostatic interactions, using the reactions and conjugates previously described [489].

### 4.2.3. siRNA as a Tool for Multimodal Cancer Treatment

An important feature of siRNA carrier systems is their ability to support multimodal therapy, which is one of the most promising strategies in cancer treatment. The synergistic effect of combining two or more treatments is more effective than using them separately, as one therapy often boosts the other. This enables lower drug doses, reducing tissue and systemic toxicity while limiting resistance to chemotherapy and radiotherapy [81,492,500]. Current multimodal approaches combined with siRNA-based gene silencing include chemotherapy, photothermal therapy (PTT), photodynamic therapy (PDT), and radiotherapy. In this context, siRNA-mediated gene silencing targets multiple cancer hallmarks, such as cell cycle progression (PLK-1, KSP), evasion of apoptosis (Survivin, Bcl-2), DNA repair (MGMT, RAD51), tumor microenvironment remodeling (CXCR4, nerve growth factor), and immune evasion (PD-L1), collectively enhancing the antitumor effect.

An example of such approaches is the use of AuNP/siRNA NPs for gene therapy/PTT [501,502], cationic photosensitizing polymers conjugated with siRNA for gene therapy/PDT [503], as well as siRNA/radionuclide/SNEEDS nanosystems for radiotherapy/gene silencing/PDT [261]. Table 4 shows some delivery systems used for this purpose.

Even when nanocarriers possess their “ideal” traits and demonstrate strong in vitro results, their success in clinical cancer treatment is not guaranteed. Several factors limit their therapeutic potential beyond laboratory conditions. These include: (i) the effects of the reticuloendothelial system and barriers like blood vessel endothelium and extracellular matrix [214]; (ii) the stability of siRNA nanocarriers and their degradation by nucleases and enzymes in the bloodstream; (iii) clearance by the mononuclear phagocytic system (MPS) and kidneys, which decreases tumor delivery [134,515]; (iv) toxicity from off-target gene silencing caused by nonspecific distribution; and (v) the challenge of targeting tumors specifically due to the tumor microenvironment’s complexity and receptor variety [134,516]. Additionally, technical issues like complex manufacturing processes that involve multiple synthesis and purification steps hamper scalability and regulatory approval. Variability between batches also poses obstacles for clinical translation compared to more controlled in vitro settings.

Some strategies for designing theranostic siRNA systems that combine effective gene silencing with the ability to monitor treatment in real time could involve functionalizing siRNA carriers with imaging agents. The nanocarrier system should respond to stimuli and act as a modular nanoplatform, allowing the incorporation of advanced imaging techniques and targeting ligand carriers [517].

In our view, the most clinically promising delivery system—offering the greatest potential to overcome the significant challenges of toxicity and immunogenicity in systemic siRNA delivery—is a sophisticated, theranostic platform that integrates several key features. This system must be capable of encapsulating siRNA (whether naked or in complexes) within its core while housing the imaging agent in its coating, ensuring maximum protection of the siRNA and high loading efficiency. Importantly, this design guarantees that the siRNA and imaging agent do not compete for space, optimizing functionality. Small inorganic nanoparticles can be used in this platform, but mainly as an additional therapeutic agent or imaging probe.

Furthermore, such platform should be able to recognize tumor cells with high internalization rates. Its size should be meticulously maintained between 100 and 200 nm, and it should be inherently “soft” to promote cellular uptake, making organic nanosystems the most promising choice. Combining lipid cores—capable of carrying other hydrophobic therapeutic agents—with biodegradable polymeric components on the surface creates a highly effective platform. These hybrid designs can incorporate pH-sensitive or membrane-disruptive features like the proton sponge effect to maximize endosomal escape, which is critical for efficacy.

A hydrophilic polymer surface minimizes undesirable corona effects and stabilizes colloidal suspension, while also allowing for a slightly positive net charge that enhances siRNA loading—even in complex form—and facilitates tumor cell uptake. This surface can be functionalized with specific groups to enable precise tumor targeting via a vector, further boosting therapeutic effectiveness.

Finally, the platform should be spheroidal, ideally a distorted sphere, as this shape ensures superior internalization and elicits a weaker immune response compared to more spherical forms. Overall, hybrid lipid/polymer organic systems stand out as the most compelling and versatile option, poised to revolutionize systemic siRNA delivery and dramatically improve clinical outcomes.

Ultimately, all variables involved in preparing a nanocarrier system need to be validated through repeated preclinical testing. Moreover, dose optimization offers the greatest potential for safe and effective systemic siRNA therapy [518].

## 5. siRNA-Targeted Genes Under Clinical Investigation

Cancer is a complex, heterogeneous disease caused by deregulated pathways that promote tumor initiation, progression, metastasis, and therapy resistance. Several candidates are currently being evaluated in clinical trials to inhibit key regulators, including serine/threonine kinases, receptor tyrosine kinases, oncoproteins, and signaling mediators. Table 5 lists representative therapeutic targets and their associated siRNA formulations in clinical trials, highlighting their biological functions, alterations in cancer, and the therapeutic benefits of targeting them. 

As shown in Table 5, siRNA-based cancer therapies in clinical trials are designed either as monotherapies or in combination strategies. These approaches include (i) inhibition of tumor survival, (ii) inhibition of angiogenesis, and (iii) induction of apoptosis [551,552]. The most common strategy in siRNA formulations is targeting proteins that act as key effectors in signaling pathways that regulate tumor growth, survival, and invasion (e.g., PLK1, PKN3, EphA2, KRAS, TGF-β1, COX-2, BCL2L12, VEGF, GSTP) [553]. Notably, many of these targets are directly linked to therapy resistance, including PLK1 (TKM-080301 candidate), KRAS (siG12D LODER candidate), TGF-β1 and COX-2 (STP-705 candidate), BCL2L12 (NU-0129 candidate), VEGF and KSP (ALN-VSP02 candidate), and GSTP (NBF-06 candidate).

In cancers, oncogene overexpression, loss of tumor suppressors, and alterations in their regulatory networks promote dedifferentiation and tumor heterogeneity [554], which decreases therapeutic effectiveness due to compensatory signaling and ultimately worsens clinical outcomes [555,556]. Significant molecular barriers include mutations or deletions in the PI3K tumor suppressor phosphatase, mutations in receptor tyrosine kinase (RTK) pathways (e.g., EGFR, HER2, MET), amplification of oncogenic signals (such as mutant K-Ras activation and SMAD4 inactivation via TGFβ), buildup of transcription factors (c-Jun, Myc, Elk, ETS, ATF), and activation of the PI3K/AKT/mTOR and MAPK/ERK pathways. For example, blocking PI3K pathway activators can trigger compensatory signaling that promotes resistance or partial response to inhibitors [556].

Due to its intrinsic heterogeneity and molecular differences among patients, it cannot be effectively treated with single-pathway monotherapies, which often lead to resistance, recurrence, activation of compensatory net [557,558]. These issues highlight the need for combination therapies that target multiple signaling pathways simultaneously [558]. For example, therapeutic candidates such as STP-075 and ALN-VSP02 demonstrate this approach by targeting distinct molecular pathways. STP-075 inhibits TGF-β1 and COX-2, while ALN-VSP02 targets VEGF and KSP simultaneously (Table 5). Such strategies are poised to enhance personalized cancer treatment by tailoring therapies to a patient’s genetic profile. Integrating genomic profiling with siRNA-based therapies offers a compelling approach in precision oncology. 

## 6. Conclusions

The increasing interest in siRNAs, driven by their success in treating various diseases including cancer, has spurred multidisciplinary research efforts. These range from using bioinformatics to design siRNAs that target specific genes to developing delivery systems that are both safe and effective for siRNA conjugates. Despite numerous clinical trials, no siRNA-based formulation has yet received approval for cancer treatment. Creating formulations that are both effective and safe for systemic cancer therapy is challenging, as it involves chemical modifications to both the siRNA and its carrier. These modifications must ensure high stability in blood, efficient cellular uptake, and successful endosomal escape of siRNA. Additionally, the system needs to be highly specific to avoid off-target effects, while minimizing toxicity and immunogenicity. Many candidates with promising in vitro results fail to reach in vivo viability due to these stringent requirements. Nonetheless, ongoing clinical trials suggest that in the near future, viable siRNA formulations for oncology will become available.

The current findings confirm that siRNA therapy holds considerable promise for cancer treatment. Evidence from combined therapies that enhance treatment outcomes, along with the development of theranostic systems for disease monitoring, indicates that siRNA as a treatment option is a promising field that will likely play an even greater role in future cancer therapies. Nevertheless, several critical challenges remain to be addressed. Ongoing research aims to streamline the production of nanosystems, enhance specificity to prevent uptake in non-target organs, and boost siRNA internalization and endosomal escape—all crucial for effective gene silencing. Although challenges remain with stability, specificity, and delivery, progress in formulation techniques, combination therapies, theranostics and patient’s genetic profile (for personalized medicine) boosts confidence that siRNAs will soon play a crucial role in precision oncology.

## Figures and Tables

**Table 1:** Some bioinformatics tools used in the design of siRNAs.

Tool	URL	Reference
DuplexFold	https://rna.urmc.rochester.edu/RNAstructureWeb/Servers/DuplexFold/DuplexFold.html	[24]
MysiRNA-Designer	https://sourceforge.net/projects/mysirna/	[25]
Genscript Package	https://www.genscript.com/sslbin/app/rnai	[19]
ThermoFisher BLOCK-It RNAi Designer	https://rnaidesigner.thermofisher.com/rnaiexpress/	[19]
siRNA design	https://www.idtdna.com/page	[19]
siDESIGN Center	https://horizondiscovery.com/en/ordering-and-calculation-tools/sidesign-center	[26]
BLAST	https://blast.ncbi.nlm.nih.gov/Blast.cgi	[26]
SMEPred	https://bioinfo.imtech.res.in/manojk/smepred/	[26]

**Table 2:** Major modifications to siRNA strands.

Modification	Advantage	Disadvantage	Application	References
**Modifications to the rest of the ribose**				
Substitution of an -OH group by less nucleophilic groups: 2′-O-Me, 2′-O-MOE, 2′-F	Increases nuclease resistance, specificity, and affinity for the target mRNA. Decreases immunogenicity	Decreases the power of the silencing	Biomolecule and small molecule conjugates	[11,31,45]
LNA training	Increases stability and specificity. Decreases toxicity	The extent and site of modification influence the physico-chemical and biological properties	Conjugates with biomolecules and small molecules	[46]
UNA training	Increases silencing potency and structural flexibility. Decreases toxicity.	Physico-chemical and biological properties depend on the extent and site of modification.	Conjugates to biomolecules and small molecules	[46,47,48]
**Modifications to the phosphate backbone**				
Substitution of a non-bridging oxygen of the phosphodiester by a sulfur to form a PS	Increases nuclease resistance, improves pharmacokinetics, and cell internalization	Efficacy and toxicity depend on the extent and site of the modification	Conjugates with biomolecules and small molecules. Incorporation into transport nanosystems	[36,51,56]
Substitution of a non-bridging oxygen by phosphate derivatives	Increases stability, reduces off-target effects and toxicity	Effectiveness depends on the extent and site of the modification	Conjugates with biomolecules and small molecules. Incorporation into transport nanosystems.	[54,58,65]
**Nucleobase modifications**				
Nucleobase modifications	Increases thermal stability, power, and selectivity	The properties can be worsened depending on the base being modified and the type of modification. Unnatural residues could be incorporated into the genome.		[11,67,69]
**Multiple modifications**				
A combination of ribose and backbone modifications	Improve biological properties and silencing efficacy.	----	----	[37,54,62]

**Table 3:** Main characteristics of LDL and HDL.

Parameter	LDL	HDL
Diameter (nm)	18–25	5–12
Density (g/mL)	1.006–1.019	1.063–1.210
Molecular weight (Da)	2.3 × 10^6^	(0.17–0.36) × 10^6^
Origin	VLDL/IDL	Liver and intestine
**Composition**		
Triglycerides (%)	10–15	2–3
Free cholesterol (%)	8–10	3–5
Cholesterol esters (%)	37–48	14–18
Phospholipids (%)	19–21	17–24
Apolipoproteins (%)	Apo B-100: (20–22)	Apo A-I, Apo A-II, Apo A-IV, Apo A-V, Apo C-I, Apo C-II, Apo C-III, Apo E, and others: (~10)

Data reported by Dai et al., 2023 [355] and specification of Lipoproteins reported by Sigma Aldrich, Burlington, MA, USA.

**Table 4:** Representative siRNA carriers targeting tumor-promoting pathways in monotherapy or combination therapy.

Carrier System	Delivery Strategy	siRNA	Effect of Gene Silencing on Cancer Signaling Pathways	Combined Therapy	Type of Cancer	Advantages	Reference
**Single treatments**							
siPD-L1@PNPs-sTN145	PLGA-based polymeric nanoparticles functionalized with sTN145 aptamer	siRNA targeting programmed cell death-ligand 1 (siPD-L1)	Disabling tumor immune evasion to enable T cell–mediated cancer destruction	-	Triple-negative breast cancer (TNBC)	siPD-L1@PNPs-sTN145 effectively delivered PD-L1 siRNA into TNBC target cells.	[504]
Chkα siRNA PEG-PEI NPs	Polymeric nanoparticles with Polyethylene glycol (PEG) conjugated polyethylenimine (PEI)	siRNA targeting Choline kinase alpha (Chkα)	Inhibition of malignant transformation and tumor progression	-	Triple negative breast cancer (TNBC)	A significant decrease in Chkα and phosphocholine was achieved in xenografts with VEGF overexpression. Demonstrated importance of tumor vascularization in achieving effective siRNA delivery	[505]
P@P EPs (PFOB/PCX/siNGF) /emulsion polyplexes (EPs)	Cationic perfluorocarbon nanoemulsions Perfluorooctyl bromide (PFOB)/Polymeric CXCR4 antagonists (PCX)/siNGF	siRNA targeting nerve growth factor	Inhibition of tumor nerve fiber formation related remodeling tumor microenvironment (TME) and promotion of the proliferation, metastasis, and chemoresistance	-	Pancreatic cancer (PC)	P@P Eps shows better tumor penetration, together with siNGF shows enhanced gene inhibition in vitro and in vivo, by inhibition of tumor nerve fiber formation reducing metastatic potential. Leading to the effective and safe suppression of tumor growth in orthotopic PC.	[506]
C12-DMA lipoplexes	Cationic lipoplexes with cholesterol/PEG/ 3,4-dimethoxyaniline lipid (DMA)	siRNA targeting Survivin	Promoted cell apoptosis	-	Breast cancer	A quaternary amine-based liposome with DMA cationic lipoplexes, successfully delivered siRNA to reduce surviving mRNA expression, indicating potential for siRNA therapeutics in breast cancer treatment.	[507]
Nanocarriers NCA_PAMAM/siRNA_ polycaprolactone-graft-poly(amidoamine) (PCL-g-PAMAM)	Polymeric dendrimer nanocarrier	siRNA targeting PLK-1 siRNA targeting PD-L1	siRNA-PLK1 blocked proliferation; siRNA-PD-L1 boosted immunity.	-	Breast cancer and colorectal cancer	The nanoconfined loading strategy enhanced the electrostatic interaction between siRNA, and the cationic moieties of PAMAM. The system improved tumor cell uptake by releasing the complex in the acidic tumor microenvironment. The system enabled effective PD-L1 silencing.	[508]
**Multimodal treatments**							
AS1411/Lipo-PTX-siPLK1	Cationic liposomes functionalized with AS1411 aptamers. It specifically binds to nucleolin, overexpressed on the surface of tumor cells.	siRNA targeting Polo-like kinase 1 (PLK-1)	Inhibition of cell cycle progression	Chemotherapy with Paclitaxel (PTX): induces mitotic arrest via microtubule targeting.	Breast cancer	Paclitaxel and PLK1-targeted siRNA using AS1411 aptamer-functionalized cationic liposomes. Functionalized with a targeting aptamer (AS1411) to further enhance targeting and tumor accumulation. The PLK-1-siRNA reduces resistance PTX. The combined therapy increased the number of apoptotic cells and reduced angiogenesis, limiting the progression of breast cancer.	[509]
PEGylated DC-Chol/DOPE	Cationic Liposomes loaded with paclitaxel	siRNA targeting kinesin spindle protein (KSP)	Cell cycle arrest during mitosis	Chemotherapy with PTX	Ovarian cancer	PEG (Polyethylene glycol) prolonged circulation time in the blood by preventing potential aggregation of cationic liposomes and avoiding nonspecific uptake by the reticuloendothelial system. DOPE (Dioleoylphosphatidylethanolamine) improved endosomal escape by destabilizing the endosomal membrane. PTX suppressed Kif15 motility, which overcame KSP resistance and enhanced the effectiveness of PTX.	[510]
RDPP(Met)/TMZ/siMGMT	Hypoxia-radiosensitive nanoparticle	siRNA targeting O6-methylguanine-DNA-methyltransferase (siMGMT)	Inhibition of DNA-damage repair	Chemotherapy with Temozolomide (TMZ) & Radiotherapy	Glioblastoma (GBM)	A hypoxia-radiosensitive nanoparticle for co-delivering TMZ and siMGMT was synthesized to sensitize chemotherapy and radiotherapy for glioblastoma (GBM). Efficient combinatorial GBM therapy. Penetrate the blood–brain barrier, target GBM cells and effectively inhibit GBM proliferation. Effective therapy for overcoming temozolomide resistance in GBM. Together enhanced the sensitivity of TMZ as well as radiotherapy.	[511]
AuPEI-HA-DOX/siRNA	Gold nanoparticles	siRNA targeting CXCR4	Inhibition of tumor microenvironment (TME) remodeling and suppression of proliferation, metastasis, and chemoresistance.	Phototherma therapy via irradiation AuNP & Chemotherapy with doxorubicin	Breast cancer	Gold nanoparticles with low-molecular weight polyethyleneimine (PEI) and hyaluronic acid (HA) transported doxorubicin (DOX) and siRNA. Combination of gene silencing, photothermal therapy and chemotherapy increased cytotoxic effect on cancer cells.	[512]
HECP2k/doxorubicin/Bcl-2 siRNA	Polymeric nanoparticles Polyethylenimine2k (PEI2k)-conjugated to hydroxyethyl cellulose (HECP2k)	siRNA targeted Bcl-2 (B-cell lymphoma 2)	Apoptosis induction	Chemotherapy with Doxorubicin	Cervical carcinoma	Endosome buffering ability of PEI2k and osmotic stress caused by HEC, facilitated cellular uptake and endosome escape, leading to efficient transfection. Induced apoptosis, enhanced chemosensitivity, high siRNA loading capacity, controlled release of siRNA. Improved tumor regression and chemosensitivity.	[513]
mHDL(CS/siRNA)	Mimetic high-density lipoproteins were prepared with Apo-A1 mimetic peptides and chitosan/siRNA complex	siRNA targeted RAD51	Inhibition of DNA repair	Radionuclide therapy with DOTA-Bombesin labeled with Actinium-225	Breast cancer	The system proved to be stable in serum, provided protection against RNases, and transfects via specific recognition by SR-B1. mHDL(CS/siRNA-RAD51) pretreatment prevented homologous repair of DBSs caused by ^225^Ac-DOTA-Bombesin, enhancing the therapeutic response.	[514]
SNEDDS (siRNA-RAD51)	Self-nanoemulsifying systems (SNEDDS) based on Phospholipon-90G, Labrafil-M1944-CS and Cremophor-RH40. In the strategy a chitosan/siRNA complex was used.	siRNA targeted RAD51	Inhibition of DNA repair to promote cancer sensitivity	Radionuclide therapy with Lutetium-177 or Photodynamic Therapy with doxorubicin irradiated with 450 nm	Breast cancer	SNEDDS (siRNA-RAD51) yielded an emulsion of 50 nm and good homogeneity (PDI 0.41) capable of efficiently transfecting siRNA-RAD51 into T47D cells. The SNEDDS(siRNA-RAD51) pretreatment in PDT or radionuclide therapy increased cell sensitivity.	[261,349]

**Table 5:** siRNA therapeutic targets in clinical trials: biological roles and effects of inhibition.

Target	Candidate	Family/Type	Main Function	Alterations in Cancer	Effect of Inhibition	Reference
**Phase II completed**						
PLK1	TKM-080301 (CTN: NCT01262235; NCT02191878)	Serine/threonine kinase (polokinase)	Regulates several phases of the cell cycle	Overexpressed in multiple cancers; associated with poor prognosis, chemo- and radioresistance	Induces apoptosis	[7,519,520,521]
PKN3	Atu027 (CTN: NCT01808638)	Protein kinase	Proliferation, migration, invasion and angiogenesis	Overexpressed in different cancers	Prevents tumor growth, metastasis and induces apoptosis	[7,12,522,523,524,525]
**Phase II in progress**						
EphA2	siRNA-EphA2-DOPC (CTN: NCT01591356)	Receptor tyrosine kinase	Proliferation, survival and differentiation	Overexpressed in several cancers; almost absent in healthy tissues; associated with high malignancy, metastasis and low survival	Inhibits carcinogenesis and angiogenesis; promotes apoptosis	[11,12,526,527,528]
KRAS G12 D mutation	siG12D LODER (CTN: NCT01676259)	Oncogene of the RAS family	Signaling for growth, differentiation, survival and apoptosis	Frequent mutations in cancer (codon 12: lung, colon, pancreas); G12D predominant in PDAC; conferring therapy resistance	Specific inhibition (e.g., G12D) blocks proliferation and tumor survival	[7,8,12,529,530,531,532]
TGF-β1, COX-2	STP705 (CTN: NCT04844983)	Cytokine (TGF-β1) Membrane enzyme (COX-2)	TGF-β1: homeostasis, apoptosis; COX-2: carcinogenesis, angiogenesis, resistance	Dysregulated TGF-β1 → epithelial–mesenchymal transition, proliferation, immune evasion; COX-2 overexpressed in aggressive tumors	Co-inhibition blocks tumor progression, angiogenesis and metastasis	[7,8,12,533,534,535,536,537,538]
**Phase I completed**						
BCL2L12	NU-0129 (CTN: NCT03020017)	Anti-apoptotic oncoprotein	Potent apoptosis inhibitor in glial cells	Overexpressed in various cancers; poor prognosis and therapy resistance	Inhibition restores apoptosis in tumor cells	[7,12,539,540,541,542]
VEGF and KSP	ALN-VSP02 (CTN: NCT01158079)	VEGF: glycoprotein RTK; KSP: microtubule-associated motor protein	VEGF: angiogenesis; KSP: mitosis and proliferation	VEGF overexpressed by tumor hypoxia → metastasis; KSP overexpressed in mitotic tumors	Co-inhibition reduces proliferation	[7,543,544,545,546,547]
**Phase I in progress**						
GSTP	NBF-06 (CTN: NCT03819387)	Detoxifying enzyme (glutathione S-transferase)	Regulation of cellular oxidation, proliferation and apoptosis	Overexpressed in aggressive tumors; contributes to therapeutic resistance	Inhibition favors apoptosis and sensitizes to therapy	[7,548,549,550]

Abbreviations: CTN: Clinical Trial Number; SNALP: Stabilized nucleic acid lipid particles; PLK1: Polo kinase type 1; GI-NET: Gastrointestinal neuroendocrine tumors; HCC: Hepatocellular carcinoma; ACC: Adrenocortical carcinoma; PDAC: Pancreatic ductal adenocarcinoma; mCRPC: Metastatic castration-resistant prostate cancer; PKN3: Protein kinase 3; PLGA: Poly (lactic-co-glycolic acid); KRAS: Kristen rat sarcoma viral oncogene homolog; EphA2: Ephrin receptor tyrosine kinase type A2; TGF-β1: Transforming growth factor type beta 1, COX-2: Cyclooxygenase 2; GSTP: Glutathione S transferase P1; CRC: Colorectal cancer; BCL2L12: gene encoding proteins containing a Bcl-2 homology domain 2; VEGF: Vascular endothelial growth factor; KSP: Kinesin spindle protein.

## Data Availability

Not applicable.
